# Cytokine/Chemokine/Growth Factor Profiles Contribute to Understanding the Pathogenesis of the Salivary Gland Dysfunction in Euthyroid Hashimoto's Thyroiditis Patients

**DOI:** 10.1155/2021/3192409

**Published:** 2021-07-11

**Authors:** K. Morawska, M. Maciejczyk, S. Zięba, Ł. Popławski, A. Kita-Popławska, J. Krętowski, A. Zalewska

**Affiliations:** ^1^Department of Restorative Dentistry, Medical University of Bialystok, 24A M. Sklodowskiej-Curie Street, 15-276 Bialystok, Poland; ^2^Department of Hygiene, Epidemiology and Ergonomics, Medical University of Bialystok, 2c Mickiewicza Street, 15-022 Bialystok, Poland; ^3^Doctoral Studies, Medical University of Bialystok, 24A M. Sklodowskiej-Curie Street, 15-276 Bialystok, Poland; ^4^Department of Radiology, Medical University of Bialystok, Poland; ^5^Department of Endocrinology, Diabetology and Internal Medicine, Medical University of Bialystok, Bialystok, Poland; ^6^Department of Restorative Dentistry and Independent Laboratory of Experimental Dentistry, Medical University of Bialystok, 24A M. Sklodowskiej-Curie Street, 15-276 Bialystok, Poland

## Abstract

Hashimoto's thyroiditis (HT) is one of the most common autoimmune diseases. It is suggested that, in addition to thyroid gland dysfunction, HT is responsible for impaired secretion from the salivary glands. The aim of this study was to evaluate the extent of symptoms of salivary gland dysfunction. We also assessed the relationship between the levels of selected cytokines, chemokines, and growth factors in unstimulated whole saliva (UWS) and the rate of UWS secretion and symptoms of xerostomia in HT patients. The study group consisted of 25 female patients diagnosed with Hashimoto's disease in its spontaneous euthyroid state who had never received hormonal treatment. In more than half of the examined patients, we observed the level of UWS secretion below 0.2 mL/min, indicating impaired secretory function of the salivary glands. Moreover, we demonstrated that the clinical symptoms of salivary gland dysfunction worsen with disease duration. Nevertheless, the inflammatory changes occurring in these glands are independent of general inflammation in the course of HT. Our results clearly indicate an abnormal profile of cytokines, chemokines, and growth factors in the UWS of HT euthyroid women as well as the fact that concentrations of IL-6 and IL-1 as well as INF-*γ*, TNF-*α*, and IL-12 may be potential biomarkers for salivary gland dysfunction in the course of HT. Furthermore, salivary IL-12 (p40) may be helpful in assessing the progression of autoimmunity-related inflammation in the course of HT. In conclusion, secretory dysfunction of the salivary glands is closely related to autoimmunity-related inflammation in the course of HT, which leads to objective and subjective symptoms of dry mouth.

## 1. Introduction

Hashimoto's disease (HT) is classified as T cell mediated and concerns the thyroid gland. It is considered as one of the most frequent autoimmune diseases [1]. It affects approximately 2% of the population and is 5 to 10 times more common in women than men [2]. HT is characterized by the presence of thyroid autoantibodies, such as thyroid peroxidase antibodies (TPO-Ab) and thyroglobulin antibodies (TG-Ab), which leads to the destruction of THE thyroid tissue. Untreated HT may result in the development of papillary thyroid cancer and thyroid carcinoma [3]. HT is also one of the factors leading to the development of hypertension, cardiovascular diseases, dyslipidemia, obesity, insulin resistance, and depression [4–6].

Moreover, salivary gland involvement in patients with HT has been described in numerous studies. Considering the role of saliva in maintaining oral health, this observation is of utmost importance for both patients and dentists [3, 7, 8]. The quantitative and qualitative deficiency of saliva is a serious problem for the patient, even more so when accompanied by other symptoms of the underlying disease. Commonly observed symptoms associated with salivary gland dysfunction include a fissured, reddened, and sore tongue, atrophic and dry oral mucosa, and increased caries incidence, mainly in the cervical region of the teeth [9, 10]. Patients report difficulty swallowing food bites and eating foods with distinct flavors as well as problems with phonation [11]. The results by Agha-Hosseini et al. [4] as well as our own research [3] demonstrated that the secretory function of the submandibular glands in HT women is impaired. This submandibular gland disturbance of function manifested significantly reduced rate of unstimulated saliva secretion (UWS). By means of objective and quantitative salivary gland scintigraphy, the secretory function of the salivary glands was found to be significantly worsened in HT patients with xerostomia (subjective sensation of dryness) compared to HT patients without xerostomia and healthy subjects with or without xerostomia [7, 12]. Syed et al. [13] argue that clinical suspicion of thyroid disease should be considered in case of a chronic reduction in salivary secretion. Therefore, these authors suggest performing a thyroid function test (hormones, antibodies) if other causes of hyposalivation have been excluded. Our own study also confirmed that both the parotid and submandibular glands of women with euthyroid HT have an impaired ability to maintain redox balance, resulting in increased oxidative modification of salivary proteins, lipids, and genetic material [3]. We associated salivary gland antioxidant dysfunction with autoimmunity-related inflammation, not with levels of thyroid hormones and TSH. Oxidative stress was not the reason for reduced salivary secretion in the course of euthyroid HT [3]. The similarity in genetic and immunopathological background between Sjögren's syndrome (SS) and HT [14–16] suggests that, by analogy with SS, the secretory function of the salivary glands in HT may have an immunological basis. It could be a consequence of impaired secretion of cytokines, chemokines, and growth factors. A decrease in the salivary gland size and increase in the inflammatory infiltrate in the salivary glands were observed in hypothyroid HT tyrosylprotein sulfotransferase knockout mice [17]. Our previous papers showed that salivary gland immunological imbalance leads to secretory dysfunction of these glands not only in the course of SS but also other autoimmune diseases, such as psoriasis [18], rheumatoid arthritis [11], and systemic sclerosis [19].

Salivary levels of selected cytokines, chemokines, or growth factors in the course of HT have not yet been determined. In the light of the aforementioned facts, such determination seems necessary in order to understand the involvement of immunological mechanisms in the development of salivary gland dysfunction in this disease. One of the aims of the presented publication was to assess the degree of salivary gland dysfunction and the occurrence of subjective and objective symptoms of salivary gland dysfunction in spontaneously euthyroid HT patients never subjected to hormonal treatment.

The study described below was further designed to assess whether or not the obtained salivary concentrations of selected cytokines, chemokines, and growth factors may be connected with the rate of UWS and symptoms of xerostomia. Furthermore, given that salivary biomarkers are used in the diagnosis of numerous systemic diseases, the secondary objective of this study was to determine whether salivary levels of cytokines, chemokines, and growth factors may present any diagnostic value when comparing the saliva of euthyroid HT female patients to that of women in the control group.

## 2. Materials and Methods

The study was approved by the Bioethics Committee of the Medical University of Bialystok (permission number: R-I-002/386/2016). Each patient and control group participant had been informed about the aims and methodology of the presented experiment and gave written consent to participate in the study.

The study group consisted of 25 female patients diagnosed with Hashimoto's thyroiditis. The diagnosis of the disease was based on a positive result of an ultrasound examination (confirming the presence of parenchymal heterogeneity) and finding blood levels of TPO-Ab and TG-Ab antibodies above the laboratory norm. The selected patients were women with euthyroid HT (free thyroxine, fT4 and thyroid stimulating hormones, TSH within the laboratory norm levels) who, importantly, had never been treated with synthetic or natural thyroid hormones or had any other treatments applied. Patients were referred for periodic check-ups to the Department of Endocrinology, Diabetology, and Internal Medicine at the Medical University of Bialystok. The control group consisted of 25 generally healthy women, matched to the study group in terms of age and BMI, who attended dental check-ups at the Department of Restorative Dentistry at the Medical University of Bialystok. The participants from the control group had normal results of thyroid ultrasound as well as blood levels of TPO-Ab, TG-Ab, fT4, and TSH in normal range for healthy individuals.

Eligibility for the study was preceded by the collection of whole blood from an ulnar vein (for hormone and antibody as well as biochemical determinations), ultrasound examination, and dental examination. Participants of the study had 10 mL samples of venous blood collected into ethylenediaminetetraacetic acid (EDTA) tubes. Blood for biochemical determinations was centrifuged (1500 × g, 4°C, 10 minutes), and the obtained plasma was stored at -84°C for no longer than 4 months, until assayed. A total of 82 female HT patients and 82 control patients were studied. 10 patients had hypothyroid HT, 25 had euthyroid HT treated with hormones, and 15 had BMI > 25. Therefore, 34 patients with HT in the state not requiring hormonal treatment and 34 control subjects of corresponding age and BMI were qualified for the study.

Serum/plasma TSH, free T4 and T3, TPO-Ab, TG-Ab, SSA/Ro-Ab, SSB/La-Ab, glucose, insulin, HOMA-IR, vitamin 25 (OH)D3, TG, and CRP were quantified by using an Abbott analyzer (Abbott Diagnostics, Wiesbaden, Germany).

On the same day, a dental examination was performed, including the measurements of DMFT (Decay, Missing, Filled Teeth) index, API (Approximal Plaque Index) index of oral hygiene, pocket probing depth (PPD), and gingival sulcus bleeding index (SBI) (Table 1). The examination was performed by one dentist (K. M.) who had obtained a suitable training beforehand, and an interrater examination was conducted in 15 randomly selected study participants with another dentist (A. Z.). The interrater reliability for DMFT was *r* = 0.97, for SBI *r* = 0.93, for API *r* = 0.96, and for PPD *r* = 95.

Based on the dental measurements, 9 HT patients were excluded from the study group due to the presence of periodontal disease and 8 from the control group because of poor oral hygiene and presence of multiple dental deposits.

HT patients had no other diseases, including autoimmune diseases, and—as noted above—their BMI was within the range indicating normal body weight (18.5–25). Both the patients and control group members were individuals with healthy periodontium and had no candidiasis and inflammation in the oral mucosa. HT patients and control subjects did not take medications on a permanent basis, including those affecting saliva secretion. The examination was performed in the second phase of the menstrual cycle, precisely between the 18th and 25th day. During 6 months preceding the research, they had not been on a weight-loss diet and had not significantly changed their lifestyle (in terms of diet, physical activity, and place of work or residence). The subjects were nonsmokers, consumed alcohol only occasionally (a glass of wine or a pint of beer at the weekend), and were not addicted to other stimulants. The clinical characteristics of patients and participants from the control group are presented in Table 2.

### 2.1. Collection of UWS

Saliva was collected one day after blood collection and dental examination, i.e., only from participants qualified for the study. UWS was collected between 7 a.m. and 9 a.m. The subjects had the collection performed on an empty stomach (last meal at least 10 hours before) and did not perform any oral hygiene procedures on this day other than rinsing the mouth with water. Saliva collection took place in a separate surgery. Upon arrival, each participant rested for 5 minutes and rinsed the mouth 5 times with water at room temperature. In a sitting position, the participant spat out saliva accumulated at the bottom of the mouth into a centrifuge tube placed in a container with ice. The saliva collection time was 15 minutes. Prior to centrifugation, the volume of obtained saliva was measured (with a calibrated pipette). This measurement allowed the rate of saliva secretion to be determined, by dividing the volume of collected saliva by the time taken to obtain it. The saliva was centrifuged for 20 minutes at 4°C, 10000 × g. Next, the supernatant was collected and frozen at -84°C for no longer than 4 months, until assayed.

To assess the prevalence of xerostomia, participants were asked to complete a questionnaire regarding the presence of dry mouth symptoms listed in the American-European classification criteria for Sjögren's syndrome [11, 20], and the Clinical Oral Dryness Score (CODS) was measured (K. M.) according to Osailan et al. [10] (Table 1). An interrater examination with another dentist (A. Z.) was conducted in 15 randomly selected study participants. The interrater reliability for CODS was *r* = 0.96.

### 2.2. Biochemical Methods

Salivary and plasma cytokines, chemokines, and growth factors were analyzed using the Bio-Plex® Multiplex System according to the manufacturer's instructions. Bio-Plex Pro Human Cytokine Assay (Bio-Rad Laboratories, Inc., Hercules, CA, USA) is a multiplex assay based on magnetic beads whose performance can be compared to a typical ELISA. Captured antibodies directed against a specific biomarker are covalently bound to magnetic beads. The coupled beads then react with the sample containing the selected biomarker. A series of rinses is performed in order to remove the unbound protein, and then a biotinylated detection antibody is added to create a sandwich complex. The final complex is formed by adding streptavidin-phycoerythrin (SA-PE) conjugate. Data from the reactions are acquired using a dedicated plate reader (Bio-Plex 200) and high-speed digital signal processor.

The concentration of total nitric oxide (NO) in saliva and plasma was determined indirectly using sulfanilamide and NEDA·2HCl (N- (1-naphthyl) ethylenediamine dihydrochloride). In this assay, stable degradation products of NO_3_^−^ were measured. Absorbance was analyzed at 490 nm [21, 22]. Total protein concentration was determined colorimetrically (Thermo Scientific Pierce BCA Protein Assay Kit, Rockford, IL, USA) using bovine serum albumin (BSA) as a standard.

### 2.3. Statistical Analysis

GraphPad Prism 8.3.0 for MacOS (GraphPad Software, Inc. La Jolla, USA) was used for statistical analysis. The normality of the distribution was assessed using the Shapiro–Wilk test. For comparison of the quantitative variables, Mann–Whitney *U* test was used. The statistical significance level was established at *p* < 0.05. The relationship between the assessed parameters was evaluated using the Spearman rank correlation coefficient. In order to determine the diagnostic utility of salivary/plasma cytokines, receiver operating characteristic (ROC) curves were drawn, and the area under the curve (AUC) was calculated. Multivariate analysis of the simultaneous impacts of many independent variables on one quantitative dependent variable was made by the means of linear regression. HT duration, TPO-Ab, TPO-AB, UWS flow, and CODS were included as independent variables. The number of subjects in the groups was determined based on our previous experiment, assuming that the power of the test was 0.9 and *α* = 0.05. The ClinCalc online calculator was used, and the minimum number of participants per group was 21.

## 3. Results

### 3.1. Stomatological Findings

The secretion of UWS by female patients with Hashimoto's disease was significantly (66%) lower than in the control group (*p* = 0.009). Moreover, 15 women from the study group had UWS level below 0.2 mL/min, which means that hyposalivation, i.e., secretory dysfunction of the salivary glands, could be confirmed in 60% of the study participants (Table 1).

Protein concentration in UWS of HT patients was significantly (56%) higher than in the control group (*p* = 0.008) (Table 1).

CODS demonstrated a significantly higher intensity of objective dry mouth symptoms in HT patients compared to the control group (*p* = 0.001). Subjective sensation of dry mouth was reported only in the group of patients with Hashimoto's disease and affected 80% of the subjects (*p* = 0.001) (Table 1).

The analysis of the results obtained during the dental examination revealed no significant differences in the DMFT, API, SBI, and PPD indices (Table 1, Table S7).

### 3.2. Saliva

#### 3.2.1. Cytokines

Significantly elevated levels of cytokines associated with Th1 lymphocyte activation (IL-3, IFN-*γ*) were found in UWS of HT patients compared to the control group (↑35%, *p* = 0.0074, ↑85% *p* ≤ 0.0001, respectively). The study group achieved considerably higher levels of interleukins connected with Th2 lymphocyte activation (IL-5, IL-6) compared to healthy controls (↑40%, *p* = 0.015, ↑54%, *p* = 0.0187, respectively). Similarly, significantly increased levels of TNF-*α* (which is a cytokine associated with Th17 lymphocyte stimulation) were observed in UWS of HT patients compared to the control group (↑93%, *p* = 0.0003). In addition, significantly higher levels of IL-12 (p40), HGF, IL-1*α*, IL-1*β*, and IL-1RA were found in UWS of study group patients compared to the control group (↑57%, *p* ≤ 0.0001, ↑38%, *p* = 0.0012, ↑38%, *p* = 0.0223, ↑54%, *p* = 0.0223, ↑34%, *p* = 0.0071, respectively). Of all cytokines tested, only salivary IL-8 and IL-10 levels reached significantly lower values in UWS of HT patients compared to healthy controls (↓21%, *p* = 0.0429, ↓69%, *p* = 0.0049, respectively). Concentrations of the other cytokines measured in UWS: IL-2, IL-2RA, IL-4, IL-7, IL-9, IL-12 (p70), IL-13, IL-15, IL-16, IL-17, IL-18, IFN-*α*2, MIF, and TNF-*β* did not differ significantly between the two groups (Table 3).

The levels of IL-18, IFN-*α*2, MIF, and TNF-*β* were not significantly different between both groups (Table 3).

#### 3.2.2. Chemokines

Concentrations of both CCL27/CTACK and CXCL1/Gro-*α* were significantly higher in UWS of HT patients compared to the controls (↑19%, *p* = 0.0195, ↑74%, *p* = 0.0005, respectively). Salivary levels of CCL2/MCP-1, CCL3/MIP-1*α*, CCL4/MIP-1*β*, *β*-NGF, CCL5/RANTES, SCF, SCGF-*β*, CCL7/MCP-3, CCL11/Eotaxin, CXCL9/MIG, CXCL10/IP-10, LIF, and CXCL12/SDF-1*α* reached similar values in both study groups (Table 4).

#### 3.2.3. Growth Factors

Concentrations of G-CSF and M-CSF in plasma of HT patients were significantly higher compared to the controls (↑29%, *p* ≤ 0.0001, ↑30%, *p* = 0.0223, respectively). The levels of the other growth factors assayed: FGF, GM-CSF, PDGF-BB, VEGF, and TRIAL reached similar values in both study groups (Table 5).

### 3.3. Plasma

#### 3.3.1. Cytokines

Plasma concentrations of cytokines responsible for Th1 lymphocyte activation (IL-3, IFN-*γ*, TNF-*β*) in HT patients were significantly elevated compared to healthy controls (↑36%, *p* ≤ 0.0001, ↑43%, *p* = 0.0001, ↑46%, *p* = 0.0028, respectively). Plasma levels of IL-5, IL-6, and IL-13 (responsible for activating Th2 lymphocytes) in the study group were considerably higher compared to the control group (↑36%, *p* ≤ 0.0001, ↑51%, *p* = 0.003, ↑26%, *p* = 0.0161, respectively). Significantly higher plasma levels of TNF-*α* (which participated in the activation of Th17 lymphocytes) were observed in HT patients compared to the controls (↑79%, *p* = 0.0001). Furthermore, plasma levels of IL-1*β*, IL-1RA, IL-2RA, IL-12 (p40), IL-12 (p70), and HGF in study group patients were significantly higher compared to the control group (↑39%, *p* = 0.017, ↑43%, *p* = 0.02, ↑9%, *p* = 0.0389, ↑28%, *p* = 0.0211, ↑49%, *p* = 0.0102, ↑28%, *p* ≤ 0.0001, respectively). Only IL-8, IL-10, and IL-17 concentrations were significantly lower in plasma of study group patients compared to the controls (↓60%, *p* ≤ 0.0001, ↓31%, *p* = 0.0004, ↓18%, *p* = 0.0466, respectively). The cytokine levels of IL-1*α*, IL-2, IL-4, IL-7, IL-9 IL-16, IL-18, IFN- *α*2, and MFI reached similar values in the plasma of both HT and control subjects (Table 3).

#### 3.3.2. Chemokines

Both CCL2/MCP-1 and SCF levels were significantly higher in the plasma of HT patients compared to the control group (↑75%, *p* = 0.0189, ↑74%, *p* ≤ 0.0001, respectively). Similarly, plasma CCL27/CTACK and LIF concentrations in the study group were significantly increased compared to healthy controls (↑101%, *p* ≤ 0.0001, ↑42%, *p* = 0.0066, respectively). Plasma levels of the chemokines CCL3/MIP-1*α*, CCL4/MIP-1*β*, *β*-NGF, CCL5/RANTES, SCGF-*β*, CCL7/MCP-3, CCL11/Eotaxin, CCL27/CTACK, CXCL1/Gro-*α*, CXCL9/MIG, and CXCL10/IP-10 did not differ significantly between the two groups (Table 4).

#### 3.3.3. Growth Factors

Concentrations of G-CSF and M-CSF in plasma of HT patients were significantly higher compared to the controls (↑29%, *p* ≤ 0.0001, ↑30%, *p* = 0.0223, respectively). The levels of the other growth factors: FGF, GM-CSF, PDGF-BB, VEGF, and TRIAL reached similar values in both study groups (Table 5).

### 3.4. NO

Concentration of NO in the UWS of HT women was significantly higher compared to the control (*p* = 0.001), whereas in plasma, it did not differ between groups (Table 2).

### 3.5. ROC Analysis

We used ROC analysis to examine the diagnostic utility of the assessed cytokines, chemokines, and growth factors. The data of ROC analysis are presented in the supplementary materials (Table S1-S6). Many biomarkers differentiate Hashimoto's patients from healthy controls with high sensitivity and specificity. However, salivary IFN-*γ*, IL-12 (p40), and TNF-*α* and plasma CTACK, SCF, G-CSF, HGF, IL-8, TNF-*α*, and IL-3 deserve special attention. Indeed, IFN-*γ* assessed in UWS with 84% sensitivity and 84% specificity distinguishes study group from healthy controls (AUC = 0.9104).

### 3.6. Multifactorial Regression Analysis

The results of multifactorial regression analysis of salivary inflammatory biomarkers are presented in Table S12. Interestingly, IL-6 and IL-1*β*levels significantly depend on HT duration, UWS flow, and CODS, while IFN-*γ* on disease duration and UWS flow. Salivary TNF-*α* statistically depends on TG-Ab and UWS flow rate, while the IL-12 level only on salivary flow (Table S12).

### 3.7. Correlations

In the group of HT patients, a negative correlation was observed between TPO-Ab levels and the rate of UWS secretion, as well as a negative correlation between salivary concentration of IL-6 and the rate of UWS secretion. Similarly, in this group, the level of salivary IL-1*α* negatively correlated with the rate of UWS secretion.

Moreover, in the study group, we found a negative correlation between the CODS and the rate of UWS secretion and a positive correlation between the CODS and the disease duration. In patients with euthyroid HT, there was a positive correlation between salivary INF-*γ* concentration and the CODS.

We demonstrated that in UWS of HT patients, IL-12 levels were positively correlated with NO concentration. Similarly, a positive correlation was observed between INF- *γ* concentration and an increase in NO level in UWS.

In the study group, we observed a positive correlation between IL-12 (p40) concentration in UWS and plasma TG-Ab concentration (Table 6).

### 3.8. Immunoassay Method Validation

Representative assay working ranges, assay sensitivity, and precision are presented in Table S13.

## 4. Discussion

Hashimoto's disease (Hashimoto's thyroiditis, HT) is a chronic autoimmune disease with complex and heterogeneous course, primarily leading to the destruction and dysfunction of the thyroid gland. However, according to available studies on the subject, it also negatively affects the function of the salivary glands, which is manifested by reduced saliva properties as well as rate of its secretion. The reason for the disturbed function of the salivary glands in the course of HT has not yet been determined. Considering the role of saliva in maintaining the health of structures present in the oral cavity as well as general well-being, explaining these pathomechanisms is of the utmost importance for both patients and doctors.

In our experiment, we narrowed down the study group to female patients who had never been treated for HT and whom did not need to include hormonal supplementation in the course of the disease. Detection of HT in most instances was accidental, e.g., during breast ultrasound or so-called periodic examinations. In such cases, it would seem that patients only require observation and prophylactic visits to an endocrinologist. However, problems of HT patients with subjective sensation of dryness in the oral cavity that appeared during dental visits, most frequently manifested by the need to drink water during the night and problems with swallowing dry food, indicated a more serious problem. These observations encouraged us to perform the present study. One of the objectives of this publication was to assess the severity of salivary gland dysfunction and the presence of subjective and objective symptoms of salivary gland impairment in spontaneously euthyroid HT patients who had never undergone hormonal treatment. Considering the involvement of immunological disturbances in the development of salivary gland disorder in the course of numerous autoimmune diseases, the study is also aimed at assessing whether the severity of salivary gland dysfunction was correlated with the concentration of selected salivary cytokines, chemokines, and growth factors. We attempted to determine whether these salivary parameters could be helpful in determining the progression of HT disease.

Since SS may be one of the causes of salivary gland dysfunction in the course of autoimmune diseases, we performed a number of diagnostic tests (Schirmer's test–data not shown, antibody determination–Table 2, questionnaire on dryness in the eyeball–data not shown–and in the oral cavity), except the labial salivary gland biopsy for which we did not obtain the consent of the Bioethics Committee. The exclusion of SS as well as other general diseases and coexisting oral inflammation led us to the conclusion that all of the observed abnormalities are due to HT.

We confirmed that the median of UWS secretion of HT patients was significantly lower compared to the control group. More importantly, 60% of them had the rate of UWS secretion below 0.2 mL/min, which is clinical evidence of salivary gland secretory insufficiency. The values of UWS < 0.2 mL/min are commonly a cutoff for assessing salivary gland dysfunction [9, 11, 23]. We demonstrated a negative correlation between TPO-Ab levels and UWS flow in the group of HT patients. This result suggests increased salivary gland secretory dysfunction with the progression of this autoimmune thyroid disease, similarly to other autoimmune diseases. In the case of SS, it was observed that increased autoimmunity, expressed by increased levels of in SSA/Lo and SSB/Ra antibodies, led to lower sensitivity or density of muscarinic receptors responsible for saliva secretion [24]. In the course of SS and another autoimmune thyroid disease—Graves' disease, elevated concentration of antimuscarinic antibodies was confirmed parallel to the progression of the disease [25, 26]. These antibodies directly inhibit the carbachol-evoked increase of intracellular calcium ions, which suggests a possible direct role of these antibodies in reducing saliva secretion [27]. Women with low UWS flow rate (below 0.2 mL/min) demonstrated the highest CODS (≥5, data not shown). The CODS is a semiquantitative clinical dry mouth score that enables the assessment of the severity and progression of oral dryness. All the female patients with a secretion rate lower than 0.2 mL/min also responded positively to all 3 questions concerning subjective symptoms of dry mouth; only 5 patients with a secretion rate of more than 0.2 mL/min responded positively to just one of the questions regarding their subjective perception of dry mouth. However, CODS values within the range of 1–4 in HT patients with UWS flow > 0.2 mL/min suggest that oral dryness symptoms may also result from changes in the coating properties of saliva, e.g., from salivary content of glycoproteins, particularly mucins, as evidenced by a positive correlation between salivary INF-*γ* concentration and the CODS in the group of HT patients. It was proven that high expression of INF-*γ* strongly correlates with reduced mucin production in acinar cells of SS patients [28]. It should undoubtedly be remembered that HT patients with UWS within the normal range and with a positive CODS test had experienced over a 50% reduction in their personal baseline UWS flow. Indeed, it is known that ≥50% reduction from personal baseline UWS flow can result in the perception of dry mouth [10]. Furthermore, we demonstrated a negative correlation between the CODS and the rate of UWS secretion in the entire group of HT patients. A negative correlation was also observed between the CODS and the disease duration, which is not surprising since objective symptoms of dry mouth result from its poor hydration or lack of cleaning effect provided by normal flow of saliva. The duration of HT in the subgroup of patients with hyposalvation was significantly longer than in the group of patients with HT with normal saliva flow (Table S8). The severity of dry mouth increases with time, as confirmed by the results of Zalewska et al. [23]. Participants from the control group reported no subjective symptoms of reduced saliva secretion, with zero values of objective dryness symptom scores (CODS = 0), which was also confirmed by the results of Osailan et al. [9].

We did not obtain similar relationships (as we described above) in the subgroup of patients with hyposalvation/normosalivation; although given the small size of this subgroup, these relationship deficiencies should be confirmed in a larger number of patients. As the ClinCalc online calculator test showed, the minimum size of the group should be 21 women.

Although it is commonly believed that HT is Th1 mediated [29], the obtained results do not indicate a dominance of either branch of the immune response. Indeed, both in plasma and UWS, a significant increase was observed in the concentrations of interleukins connected with the activation of Th1 lymphocytes (IFN-*γ* ↑88% in saliva and ↑43% in plasma; IL-2RA ↑9% and TNF-*β* ↑46% in plasma), Th2 (IL-5 and IL-6 ↑40% and ↑54%, respectively, in saliva and ↑51% and ↑65%, respectively, in plasma; IL-13 ↑26% in plasma), or Th17 (IL-6 and TNF-*α* ↑54% and ↑93%, respectively, in saliva and ↑65% and ↑79%, respectively, in plasma) with reduced secretion of Th2 cytokines (IL-10 ↓69% in saliva and ↓17% in plasma) [30]. We further observed increased salivary and plasma concentrations of IL-1*α*, IL-1*β*, IL-1RA, IL-3, IL-12 (p40), and plasma IL-12 (p70). Many of the tested cytokines did not differ between the study and control groups, both in saliva and plasma. Taking into account the subgroups of HT patients, we observed significantly higher levels of IFN-*γ*, IL-6, IL-12 (p40), IL1*α*, IL-1RA, IL-4, IL-7, and lower IL-10 concentration in the UWS of HT patients with hyposalivation compared to normal salivation HT patients (Table S9). However, we would like to emphasize that these results should be interpreted very carefully due to the insufficient number of cases in the subgroups.

We noted a significant increase in the concentration only of some salivary chemokines (CCL27/CTACK, CXCL1/Gro-*α*) and found no correlation between the concentrations of the assayed chemokines and interleukins. When analyzing the concentration of chemokines in the subgroups of HT patients, we did not find significant differences (Table S10). As leukocyte migration is the dominant biological process regulated by chemokines, the obtained results are highly likely to demonstrate enhanced migration of white blood cells from blood vessels into the salivary gland parenchyma [31]. It was shown that enlargement of the thyroid gland in the course of HT is accompanied by significantly increased blood flow, vascularization of this gland, and vascular permeability due to boosted expression of VEGF [32]. VEGF is a recognized angiogenic factor and enhancer of vascular permeability [33]. It is not known whether similar morphological changes occur in the salivary glands as in the thyroid gland. The significant increase of salivary VEGF levels may prove increased permeability of salivary gland blood vessels. Interesting but not surprising overall, higher VEGF concentrations were noted in the UWS of patients with hyposalivation compared to patients with HT and without objective salivary gland dysfunction (Table S11). As mentioned above, we observed increased salivary concentrations of IL-1*α*, IL-1*β*, IL-1RA, and IL-12, which can be derived from inflammatory cells infiltrating the salivary glands. It is well known that these cytokines are released by incoming stimulated monocytes, activated macrophages, and vascular endothelial cells. Previous studies demonstrated that elevated level of salivary IL-6 also correlates significantly with the degree of lymphocytic infiltration in the labial salivary glands of SS patients [34, 35], implicating the role of this cytokine in SS progression [35]. In the presented experiment, we did not perform labial gland biopsy. Spearman rank correlation coefficient showed the positive correlations between salivary IL-6 and UWS flow, IL-1*α*, and UWS flow as well as INF-*γ* and the CODS. Multifactorial regression analysis of salivary inflammatory biomarkers revealed that IL-6 and IL-1*β* level significantly depend on HT duration, UWS flow, and CODS, while IFN-*γ* on disease duration and UWS flow. Salivary TNF-*α* statistically depends on TG-Ab and UWS flow rate, while the IL-12 level only on salivary flow. Hypothesizing, these results suggest that increased levels of IL-6, IL-1*α*, and INF-*γ* in UWS may be related to the level of salivary gland degeneration, leading to reduced saliva secretion. It has been evidenced that IL-6 and IL-1 as well as INF-*γ*, TNF-*α*, and IL-12 play an important role in the destruction of salivary gland tissue [36–38]. INF-*γ* not only enhances remodeling of the extracellular space of salivary glands through upregulation the production of metalloproteinases [39] but, similar to TNF-*α* and IL-12, activates intrinsic apoptosis pathways [37, 38, 40], and IL-6 and IL-1 boost apoptotic damage by exposing cytosolic autoantigen present in HT [41]. IL-6 also significantly intensifies the local inflammatory process by inducing T cell proliferation and B-lymphocyte differentiation and decreasing the number of Treg cells [42, 43]. Moreover, it has been proven that increased secretion and concentration of IL-1, TNF-*α*, or INF-*γ* in the inflamed salivary gland microenvironment may inhibit acetylcholine release, resulting in an attenuated acinar cell response and reduced saliva secretion [44]. Furthermore, it is well established that NO controls the secretion of saliva. NO has been demonstrated to exert a stimulatory effect on salivation in normal male rats [45], whereas excess NO is detrimental to numerous cells, including acinar cells and cells of ducts within the salivary glands, causing apoptosis of these cells and acute impairment of salivary gland function [37]. In the presented study, we observed that increased salivary IL-12 and INF-*γ* levels correlated positively with elevated salivary NO concentration, indicating that IL-12 and INF-*γ* may participate in the inhibition of saliva secretion by the NO-mediated pathway. The obtained results were not reported in previous studies, and their interpretation is a far-reaching assumption; therefore, further studies are necessary to assess the utility of IL-6 and IL-1 as well as INF-*γ*, TNF-*α*, and IL-12 as biomarkers for salivary gland dysfunction in the course of HT.

The limitation of the current publication is the small number of women with HT; however, as the ClinCalc online calculator shows, the number of 25 is sufficient for the analysis. However, these are selected patients: without a history of hormone therapy, with a healthy periodontium, no other inflammatory diseases of the oral cavity, and no other diseases, including autoimmune diseases. The composition of saliva depends on the local condition in the oral cavity and the general health of the patient. Therefore, in order to assess the function of the salivary glands due to HT, patients should be very critically selected, which could results in a small group size.

In the current publication, we evaluated the utility of the assayed parameters for the diagnosis of HT. ROC analysis showed that some of the studied parameters can be helpful in differential diagnosis of female patients with HT in an untreated euthyroid state from healthy women matched by age and BMI (Table S1, S2, S3). Special attention should be paid to salivary INF-*γ* and IL-12 (p40) levels that distinguish the study group from the controls (AUC = 0.91, *p* < 0.0001, AUC = 0.86, *p* < 0.0001, respectively). Moreover, salivary IL-12 (p40) concentration correlated positively with plasma TG-Ab, which may suggest that changes in salivary IL-12 (p40) concentrations reflect the progression of Hashimoto's disease. Changes in the levels of the studied parameters do not reflect the changes observed in plasma. Moreover, we observed no correlation between the concentrations of the examined parameters in UWS and plasma. The lack of such correlations proves that inflammatory changes and the related salivary gland dysfunction are independent of general inflammation in the course of HT. Similarly, ROC analysis revealed that plasma proteins, different from those present in UWS, could be helpful in the diagnosis of HT (CTACK: AUC = 0.9, *p* < 0.0001, IL-3: AUC = 0.82, *p* < 0.0001, G-CSF: AUC = 0.87, *p* < 0.0001, HGF: AUC = 0.97, *p* < 0.0001, IL-8: AUC = 0.89, *p* < 0.0001, TNF-*α*: AUC = 0.84, *p* < 0.0001). However, despite the promising results of ROC analysis and given the low number of female HT patients participating in the experiment, this analysis should be tested on a wider population of euthyroid HT patients.

## 5. Conclusions

1. The reduction of UWS secretion in female patients with euthyroid HT compared to the controls is a manifestation of impaired function of the salivary glands. Moreover, UWS flow values below 0.2 mL/min, observed in 60% of the HT patients participating in the experiment, are clinical evidence of secretory dysfunction of the salivary glands
2. The severity of salivary gland secretory dysfunction is closely associated with autoimmunity-related inflammation in the course of HT
3. Clinical symptoms of salivary gland dysfunction worsen with disease duration
4. We demonstrated impaired profiles of cytokines, chemokines, and growth factors in the UWS and plasma of euthyroid HT women
5. The evaluation of the levels of assayed cytokines in the saliva and plasma of patients with untreated euthyroid HT does not indicate the dominance of any of the branches of the immune response
6. IL-6 and IL-1 as well as INF-*γ*, TNF-*α*, and IL-12 may be potential biomarkers for salivary gland dysfunction in the course of HT
7. Inflammatory changes and the associated dysfunction of the salivary glands are independent of general inflammation in the course of HT
8. Salivary IL-12 (p40) may be helpful in assessing the progression of autoimmunity-related inflammation in the course of HT

## Figures and Tables

**Table 1:** Stomatological characteristics of patients and control group participants (UWS: unstimulated saliva; TP: total protein; CODS: Clinical Oral Dryness Score; DMFT: Decayed, Missing, Filled Teeth; API: approximal plaque index; SBI: sulcus bleeding index, PPD: periodontal pocket depth; NS: statistically insignificant).

	Control, *n* = 25	HT, *n* = 25	*p*
UWS mL/min	0.67 (0.46–0.89)	0.27 (0.1–0.61)	<0.0001
No. of women (%) with hyposalivation (UWS < 0.2 mL/min)	0 (0)	15 (60)	<0.00001
TP NWS (mg/mL)	895 (658.23–1250.1)	1400 (1002.1-1600.56)	<0.0001
CODS (1–10)	0	6 (1–10)	<0.0001
No. of women with CODS ≥ 5	0	13	<0.0001
No. of women with CODS ≥ 1 ≤ 4	0	8	<0.001
Subjective oral dryness, *n* (%)	0 (0)	20 (80)	<0.0001
DMFT	18 (15–25)	16 (14–25)	NS
API	24.56 (0–30)	21.54 (0–32)	NS
SBI	0.45 (0–1)	0.33 (0–1)	NS
PPD (mm)	2.0 (0.5–2.5)	2.0 (0.5–2.5)	NS

**Table 2:** Clinical characteristics of the participants of the study (HT: Hashimoto patients; BMI: body mass index; TSH: thyroid-stimulating hormone; TPO-Ab: thyroid peroxidase antibody; TG-Ab: thyroid peroxidase antibody; SSA/Ro-Ab: anti-Sjögren's syndrome type A antibody; SSB/La-Ab: anti-Sjögren's syndrome type B antibody; HOMA-IR: homeostatic model assessment index; TG: triglyceride; NO: nitric oxide; UWS: unstimulated whole saliva; ND: not detectable; NS: statistically insignificant).

	Control, *n* = 25 M (min-max)	HT, *n* = 25 M (min-max)	*p*
Age (years)	34.3 (27.2–42)	34.5 (27.8–41.5)	NS
BMI (kg/m^2^)	20.5 (19.35–23.96)	21.7 (19.2–24.61)	NS
TSH (*μ*U/mL)	1.23 (0.48–2.3)	1.56 (0.7–2.99)	NS
Free T4 (ng/mL)	1.36 (1.1–1.39)	1.26 (0.76–1.62)	NS
Free T3 (pg/mL)	2.26 (1.75–3.96)	2.86 (2.63–4.51)	NS
TPO-Ab (IU/mL)	0.5 (0.36–2.51)	625.6 (132.4–768)	<0.0001
TG-Ab (IU/mL)	0.4 (0.23–1.91)	318.3 (145.8–437)	<0.0001
SSA/Ro-Ab	ND	ND	
SSB/La-Ab	ND	ND	
Glucose (mg/dL)	79.56 (72.35–85.01)	83.01 (73.56–88.05)	NS
Insulin (mU/mL)	4.25 (3–11.2)	3.89 (3–10.9)	NS
HOMA-IR	0.89 (0.53–2.41)	0.8 (0.51–2.39)	NS
Witamin 25-OH D3 (ng/mL)	47 (32–51.1)	42.98 (30–50)	NS
TG (mg/dL)	52 (39–79.56)	49 (36–85.21)	NS
CRP mg/L	0.4 (0.2–0.8)	0.63 (0.23–0.92)	NS
HT duration (years)	0	5.8 (3.8–8.3)	<0.0001
Thyroid gland's nodules	ND	ND	
NO UWS (*μ*mol/mg protein)	0.15 (0.01-0.08)	0.26 (0.02–0.56)	0.0011
NO plasma (*μ*mol/mg protein)	0.09 (0.014- 0.19)	0.08 (0.016–0.24)	0.9082

**Table 3:** Effect of Hashimoto's disease on cytokine levels in saliva and plasma. IL: 1*α*, 1*β*, 1RA, 2, 2RA, 3,4, 5,6,7,8,9, 10, 12 (p40), 12 (p70), 13, 15, 16, 17, 18-interleukin: 1*α*, 1*β*, 1RA, 2, 2RA, 3,4, 5,6,7,8,9, 10, 12 (p40), 12 (p70), 13, 15, 16, 17, 18; IFN-*γ*: interferon-*γ*; IFN-*α*2: interferon-*α*2; TNF-*α*, *β*: tumor necrosis factor *α*, *β*; HGF: hepatocyte growth factor; MIF: macrophage migration inhibitory facto;, ND: no detectable.

	Saliva					Plasma				
	Control group		Study group		*p* value	Control group		Study group		*p* value
	Mean ± SD	Median (min-max)	Mean ± SD	Median (min-max)		Mean ± SD	Median (min-max)	Mean ± SD	Median (min-max)	
IL-3	35.11 ± 11.41	38.88 (7-56)	47.33 ± 17.3	45 (13-80)	0.0074	26.72 ± 4.009	27.47 (19.79-35.24)	36.28 ± 9.588	36.01 (19.1-58.72)	<0.0001
IFN-*γ*	25.12 ± 8.914	23.57 (12.23-54.5)	46.5 ± 15.09	43.54 (19.11-81.46)	<0.0001	164.4 ± 50.86	169.5 (43.5-236)	235.9 ± 69.13	233.2 (123-427.9)	0.0001
IL-5	6.065 ± 1.85	6.05 (0.6-9.4)	8.49 ± 3.975	8.6 (1.2-15.65)	0.015	106.4 ± 75.49	94.43 (7.968-320.2)	160.3 ± 67.12	158.5 (41.24-301.3)	0.003
IL-6	72.01 ± 27.06	73.59 (19-136)	110.8 ± 69.45	103 (17-297)	0.0187	3.065 ± 0.6913	3.147 (1.568-4.517)	5.051 ± 2.172	5.202 (0.7955-9.66)	0.0002
TN-*α*	8.173 ± 5.213	6.975 (0.625-21.68)	15.79 ± 8.211	15.15 (1.28-37.39)	0.0003	12.5 ± 5.202	12.63 (6.019-27.13)	22.32 ± 8.808	21.27 (6.815-38.84)	<0.0001
IL-12 (p40)	12.45 ± 4.186	12 (6.614-21.75)	19.5 ± 5.242	19.5 (8.004-29.9)	<0.0001	13.61 ± 3.629	13.81 (8.131-21.28)	17.41 ± 6.56	17.59 (6.718-33.75)	0.0211
HGF	3.908 ± 0.9919	3.842 (1.599-6.273)	5.412 ± 1.614	5.373 (3.063-8.694)	0.0012	60.7 ± 6.446	60.69 (43.81-74.71)	77.82 ± 9.324	75.83 (66.35-103.7)	<0.0001
IL-1*α*	760.6 ± 360.9	766.1 (189.2-1375)	1054 ± 441.2	1148 (408.2-2182)	0.0223	328 ± 227.5	271 (15-970)	458.5 ± 346	417.5 (39.38-1619)	0.0741
IL-1*β*	99.04 ± 65.47	74.05 (22.53-245.4)	153.2 ± 86.18	153.3 (28.93-343.5)	0.0223	17.54 ± 9.707	17.28 (1.302-33.61)	24.46 ± 8.32	24.38 (8.663-48.13)	0.017
IL-1RA	5170 ± 1847	5071 (917-7936)	6951 ± 2239	6707 (3196-10575)	0.0071	13.49 ± 7.875	12.01 (0.7689-31.98)	19.32 ± 8.643	17.87 (4.071-40.51)	0.02
IL-8	1564 ± 913.3	1479 (87.75-3855)	1230 ± 1167	882 (21-5426)	0.0429	24.43 ± 8.201	25.36 (6.595-38.56)	9.871 ± 7.801	7.724 (-5.24-27.19)	<0.0001
IL-10	28.49 ± 10.39	26.5 (10.22-47)	19.69 ± 9.166	19 (2.235-40.22)	0.0049	34.42 ± 9.252	35.14 (13.04-49.94)	23.61 ± 11.09	21.76 (4.953-47.42)	0.0004
IL-2	27.58 ± 10.46	30 (4-46)	30.23 ± 12.54	29 (9.218-58)	0.6685	116.7 ± 48.75	129.7 (18.51-189.3)	140.9 ± 64.07	144.7 (25.42-298.6)	0.1213
IL-2RA	39.98 ± 20.56	37.13 (3.823-86.66)	40.09 ± 20.86	39 (2.598-87)	0.9578	28.35 ± 3.504	27.91 (22.57-34.75)	30.79 ± 5.045	32.49 (19.24-37.89)	0.0389
IL-4	16.22 ± 7.095	15 (2-33.5)	19.85 ± 7.338	18 (9-36)	0.0625	23.97 ± 4.79	24.39 (15.87-31.69)	26.25 ± 11.21	26.8 (1.017-47.67)	0.3067
IL-7	17.91 ± 4.275	18 (9.391-25)	17.8 ± 8.887	16.5 (4.807-42)	0.455	5.099 ± 2.223	4.495 (1.972-10.08)	7.364 ± 4.663	6.964 (0.6749-17.65)	0.1035
IL-9	45.98 ± 17.78	47 (16.48-82)	53.85 ± 21.96	55 (12.07-99)	0.1806	49.19 ± 20.28	48.04 (21.43-91.61)	55.94 ± 25.79	58.96 (8.061-92.59)	0.2799
IL-12 (p70)	22.59 ± 8.404	22.79 (3.101-36.6)	25.25 ± 7.734	24 (9.988-36.5)	0.2907	16.17 ± 6.58	16.96 (3.493-30.17)	24.02 ± 11.11	20.41 (2.664-44.17)	0.0102
IL-13	1.013 ± 0.4813	1.027 (0.3138-1.833)	1.287 ± 0.7352	1.137 (0.1965-2.41)	0.2022	9.829 ± 4.507	9 (3.549-21.14)	12.41 ± 3.768	12 (5.41-22)	0.0161
IL-15	10.72 ± 5.484	10.2 (0.473-23)	12.35 ± 9.511	9.6 (2.85-35.3)	0.7619	ND	ND	ND	ND	ND
IL-16	981.3 ± 515.6	941.6 (80.27-2125)	1095 ± 594.9	1012 (235.8-2163)	0.5253	161.6 ± 127.1	129 (11.15-587)	175.3 ± 165.5	141.5 (5.34-780)	>0.9999
IL-17	ND	ND	ND	ND	ND	33.74 ± 11.03	29.5 (21.41-58)	27.58 ± 12.11	26.6 (8.334-56.7)	0.0466
IL-18	369.3 ± 379.1	331.5 (10.73-1999)	399.7 ± 403.2	280 (4.705-1829)	0.9847	20.7 ± 9.127	22.17 (3.895-33.62)	23.2 ± 14.99	21.47 (2.131-57.12)	0.6581
IFN-*α*2	4.61 ± 0.9551	4.539 (2.918-7.328)	5.053 ± 1.844	5.054 (1.601-10.47)	0.3859	22.81 ± 6.25	22.29 (9.482-34.99)	22.71 ± 7.463	21.31 (2.789-39.93)	0.8777
MIF	759.7 ± 323.3	661 (258.2-1424)	750.5 ± 538.5	601 (119.5-2221)	0.4185	196.5 ± 78.99	208 (44.66-340.8)	206.2 ± 79.61	184.4 (81.59-359.2)	0.8929
TNF-*β*	5.702 ± 1.994	5.986 (0.7912-9.791)	7.135 ± 2.727	7.033 (1.758-11.87)	0.0925	6.692 ± 2.137	6.997 (1.33-10.08)	9.793 ± 4.96	10.01 (-2.785-17.23)	0.0028

**Table 4:** Effect of Hashimoto's disease on chemokine levels: CCL27/CTACK: chemokine ligand 3/monocyte chemoattractant protein*-*1; CCL3/MIP-1-*α*: chemokine ligands 3/macrophage inflammatory protein*1*-alpha; CCL4/MIP-1 *β*: chemokine ligands 4/macrophage inflammatory protein*1 β*; *β* -NGF: *β*-nerve growth factor; CCL5/RANTES: chemokine ligand 5/regulated on activation, normal T cell expressed and secreted; SCF: stem cell factor; SCGF-*β*: stem cell growth factor-*β*; CCL7/MCP-3: chemokine ligand 7/monocyte-chemotactic protein *3*; CCL11/eotaxin: chemokine ligand 11/eatoksin; CCL27/CTACK: chemokine ligand 27/cutaneous T cell-attracting chemokine; CXCL1/GRO-*α*: chemokine (C-X-C motif) ligand 1/growth-regulated oncogene-alpha; CXCL9/MIG: chemokine (C-X-C motif) ligand 9/monokine induced by gamma interferon; CXCL10/IP-10: chemokine (C-X-C motif) ligand 10/interferon gamma-induced protein *10;* LIF: leukemia inhibitory factor; CXCL12/SDF-1 *α*: chemokine (C-X-C motif) ligand 12/stromal cell-derived factor 1; ND: no detectable.

	Saliva					Plasma				
	Control group		Study group		*p* value	Control group		Study group		*p* value
	Mean ± SD	Median (min-max)	Mean ± SD	Median (min-max)		Mean ± SD	Median (min-max)	Mean ± SD	Median (min-max)	
CCL27/CTACK	17.80 ± 5.689	18.08 (8.970-33.69)	21.23 ± 6.034	20.27 (11.53-36.62)	0.0195	70.22 ± 15.1	72.93 (39.18-111.6)	141.2 ± 71.73	128.3 (60-375.5)	<0.0001
CXCL1/Gro-*α*	8545 ± 3904	8235 (1768 -15622)	14898 ± 6761	15456 (3393-30180)	0.0005	17772 ± 7093	18731 (3763-33741)	19882 ± 7155	21785 (4134-29760)	0.2092
CCL2/MCP-1	703 ± 360.5	707.8 (198-1490)	727.7 ± 618.5	677 (24.52-2896)	0.7583	4.51 ± 2.51	4.557 (0.2395-9.026)	7.915 ± 4.844	5.865 (1.748-19.22)	0.0189
CCL3/MIP-1*α*	33.89 ± 22.89	28 (5.895-118.5)	44.79 ± 67.19	28 (7.238-354)	0.7914	123.6 ± 71.94	120.1 (11.45-268.1)	141.2 ± 92.17	141.4 (5.762-375.2)	0.5768
CCL4/MIP-1*β*	63.07 ± 26.34	54.5 (27.5-138)	61.73 ± 31.01	63 (2.401-129.5)	0.8362	124 ± 79.66	117 (16.15-276.7)	136.5 ± 87.9	140.4 (16.26-332.6)	0.6721
*β*-NGF	62.03 ± 23.58	66 (8.513-116.3)	70.33 ± 36.36	61 (8.457-143.3)	0.4973	84.27 ± 48.49	82.24 (6.667-179.1)	103.6 ± 52.8	99.81 (33.11-275.2)	0.263
CCL5/RANTES	2.94 ± 1.038	2.927 (0.394-4.531)	3.48 ± 1.46	3.201 (1.084-6.698)	0.2218	1550 ± 620.2	1470 (519.6-2704)	1978 ± 915.4	1740 (465.3-4047)	0.0916
SCF	77.67 ± 39.69	72 (5.96-150)	89.36 ± 79.58	76 (6.221-314)	0.7914	32.98 ± 9.754	33.06 (17.22-50.29)	57.39 ± 19.22	55.61 (27.62-89.57)	<0.0001
SCGF-*β*	63.07 ± 26.34	54.5 (27.5-138)	61.73 ± 31.01	63 (2.401-129.5)	0.8362	539.4 ± 316.7	560.9 (43.81-1213)	660.2 ± 270.4	658.4 (150.4-1260)	0.2467
CCL7/MCP-3	18.04 ± 5.184	18 (9.874-30)	20.1 ± 5.504	19.5 (10.93-30.9)	0.1931	639.5 ± 136.9	619.5 (275.5-879.5)	697.3 ± 195.3	731.6 (206.5-952.3)	0.1077
CCL11/Eotaxin	20.05 ± 6.526	19.99 (8.792-37.74)	21.89 ± 9.195	18.03 (9.54-43.23)	0.8475	72.15 ± 22.91	68.86 (15.32-120.1)	82.09 ± 32.24	75.98 (39.9-159.7)	0.441
CXCL9/MIG	608.7 ± 655.1	333.5 (17.5-3001)	1090 ± 1552	542.5 (74.97-7798)	0.3254	101.1 ± 92.73	68.06 (4.366-413.3)	133.7 ± 72.15	144 (18.91-307.8)	0.0568
CXCL10/IP-10	6686 ± 3147	6608 (1730-12452)	9053 ± 5665	7868 (833.7-23044)	0.1954	566.8 ± 346.5	542 (12.02-1490)	742.9 ± 648.9	574. (12.51-2896)	0.444
LIF	20.42 ± 10.76	19 (2.161-45.5)	25.68 ± 13.15	23 (8.214-53.5)	0.2329	24.86 ± 9.771	24.31 (9.054-42.78)	35.38 ± 16.87	35.87 (2.65-71.33)	0.0066
CXCL12/SDF-1*α*	53.34 ± 18.2	55 (17.55-91.5)	56.4 ± 22.92	53.48 (13.95-101)	0.6756	ND	ND	ND	ND	ND

**Table 5:** Effect of Hashimoto's disease on growth factor levels: FGF: fibroblast growth factor; G-CSF: granulocyte colony-stimulating factor; GM-CSF: granulocyte-macrophage colony-stimulating factor; M-CSF: macrophage colony-stimulating factor; PDGF-BB: platelet-derived growth factor–BB; VEGF: vascular endothelial growth factor.

	Saliva					Plasma				
	Control group		Study group		*p* value	Control group		Study group		*p* value
	Mean ± SD	Median (min-max)	Mean ± SD	Median (min-max)		Mean ± SD	Median (min-max)	Mean ± SD	Median (min-max)	
G-CSF	493.9 ± 72.24	497 (363.1-649.2)	574.1 ± 115.8	587.8 (344.1-894.8)	0.0066	603.7 ± 91.3	590.2 (454.4-764.7)	777.1 ± 164	726.8 (455.9-1162)	<0.0001
VEGF	1443 ± 850.1	1221 (53.24-3643)	2631 ± 1480	2433 (634.8-6122)	0.0025	191.1 ± 64.73	179.4 (91.19-331)	271.3 ± 158.2	252.3 (24.03-608.9)	0.0842
TRIAL	519.3 ± 307.7	441.3 (16.85-1154)	1009 ± 1011	727.1 (24.71-4653)	0.0472	80.32 ± 30.77	78.42 (26.96-143.5)	101.6 ± 47.97	101.7 (12.02-191.1)	0.0878
FGF	5.371 ± 1.205	5.412 (3.418-8.347)	5.737 ± 1.197	5.54 (3.717-8.09)	0.3571	34.45 ± 7.104	33.35 (19.34-46.92)	33.85 ± 9.314	31.99 (17.48-54.19)	0.6443
GM-CSF	24.12 ± 6.793	22.65 (11.77–37.98)	25.6 ± 10.25	25.17 (5.405 –50.55)	0.5637	98.28 ± 15.9	100.2 (70.8-140.6)	102.9 ± 26.21	103.9 (48.13-146.8)	0.4762
M-CSF	228.5 ± 137.5	206 (13.03-582.5)	309.7 ± 268.6	238 (18.8-1301)	0.2182	65.97 ± 30.89	69.24 (4.661-135.7)	85.52 ± 34.76	98.87 (5.619-131.4)	0.0223
PDGF-BB	480.9 ± 165.9	490 (103.6-740)	534 ± 262.3	548.2 (111-1060)	0.5096	1041 ± 337.1	1092 (351.6-1689)	976.5 ± 552	1095 (39.4-2135)	0.6305

**Table 6:** Statistically significant correlations in the study group (TG-Ab: thyroid peroxidase antibody; UWS: unstimulated saliva; IL-1*α*: interleukin 1*α*; IL-6: interleukin 6; IL-12: interleukin 12; INF-*γ*: interferon *γ*; CODS: Clinical Oral Dryness Score, NO: nitric oxide; IL-12 (p40): interleukin 12 (p40); TG-A: thyroid peroxidase antibody).

Pairs of variables	*r*	*p*
TPO-Ab and UWS flow	-0.865	<0.0001
IL-6 and UWS flow	-0.951	<0.0001
IL-1*α* and UWS flow	-0.864	<0.0001
CODS and UWS flow	-0.885	<0.0001
CODS and duration of Hashimoto's disease	0.751	<0.0001
IFN-*γ* and CODS	0.488	0.013
IL-12 and NO	0.723	<0.0001
INF-*γ* and NO	0.442	0.027
IL-12 (p40) UWS and plasma TG-A	0.557	0.004

## Data Availability

The datasets generated for this study are available on request to the corresponding author.

## Supplementary Materials

Supplementary Materials Table S1: receiver operating characteristic (ROS) analysis of salivary cytokines in patients with Hashimoto's disease and healthy controls. Table S2: receiver operating characteristic (ROS) analysis of plasma cytokines in patients with Hashimoto's disease and healthy controls. Table S3: receiver operating characteristic (ROS) analysis of salivary chemokines in patients with Hashimoto's disease and healthy controls. Table S4: receiver operating characteristic (ROS) analysis of plasma chemokines in patients with Hashimoto's disease and healthy controls. Table S5: receiver operating characteristic (ROS) analysis of salivary growth factors in patients with Hashimoto's disease and healthy controls. Table S6: receiver operating characteristic (ROS) analysis of plasma growth factors in patients with Hashimoto's disease and healthy controls. Table S7: stomatological characteristics of the HT subgroups. Table S8: clinical characteristics of the participants of the HT subgroups. Table S9: differences in salivary inflammatory profile in HT subjects with hyposalivation compared to normal salivary secretion. Table S10: differences in salivary chemokine profile in HT subjects with hyposalivation compared to normal salivary secretion. Table S11: differences in salivary growth factor profile in HT subjects with hyposalivation compared to normal salivary secretion. Table S12: multifactorial regression of selected salivary biomarkers in all enrolled patients. Table S13: LLOQ, ULOQ, LOD, and intra- and interassay precision %CV.
