# Supplementary material for: Cytokine/Chemokine/Growth Factor Profiles Contribute to Understanding the Pathogenesis of the Salivary Gland Dysfunction in Euthyroid Hashimoto's Thyroiditis Patients
Source: Mediators Inflamm. 2021 Jul 11;2021:3192409. doi: 10.1155/2021/3192409 (PMC8289575; doi:10.1155/2021/3192409)
Supplement: Supplementary Materials — Table S1: receiver operating characteristic (ROS) analysis of salivary cytokines in patients with Hashimoto's disease and healthy controls. Table S2: receiver operating characteristic (ROS) analysis of plasma cytokines in patients with Hashimoto's disease and healthy controls. Table S3: receiver operating characteristic (ROS) analysis of salivary chemokines in patients with Hashimoto's disease and healthy controls. Table S4: receiver operating characteristic (ROS) analysis of plasma chemokines in patients with Hashimoto's disease and healthy controls. Table S5: receiver operating characteristic (ROS) analysis of salivary growth factors in patients with Hashimoto's disease and healthy controls. Table S6: receiver operating characteristic (ROS) analysis of plasma growth factors in patients with Hashimoto's disease and healthy controls. Table S7: stomatological characteristics of the HT subgroups. Table S8: clinical characteristics of the participants of the HT subgroups. Table S9: differences in salivary inflammatory profile in HT subjects with hyposalivation compared to normal salivary secretion. Table S10: differences in salivary chemokine profile in HT subjects with hyposalivation compared to normal salivary secretion. Table S11: differences in salivary growth factor profile in HT subjects with hyposalivation compared to normal salivary secretion. Table S12: multifactorial regression of selected salivary biomarkers in all enrolled patients. Table S13: LLOQ, ULOQ, LOD, and intra- and interassay precision %CV. [file 3192409.f1.docx]

|  | Saliva | | | | | | | |
| --- | --- | --- | --- | --- | --- | --- | --- | --- |
|  | AUC | 95% confidence interval | P value | Cut Off | Sensitivity% | 95% CI | Specificity% | 95% CI |
| **IL-3** | 0.7184 | 0.5741 to 0.8627 | 0.0081 | < 41.30 | 68 | 48.41% to 82.79% | 64 | 44.52% to 79.75% |
| **IFN-γ** | 0.9104 | 0.8281 to 0.9927 | <0.0001 | < 32.52 | 84 | 65.35% to 93.60% | 84 | 65.35% to 93.60% |
| **IL-5** | 0.6992 | 0.5465 to 0.8519 | 0.0157 | < 6.442 | 64 | 44.52% to 79.75% | 64 | 44.52% to 79.75% |
| **IL-6** | 0.6928 | 0.5414 to 0.8442 | 0.0194 | < 81.50 | 64 | 44.52% to 79.75% | 60 | 40.74% to 76.60% |
| **TNF-α** | 0.7904 | 0.6604 to 0.9204 | 0.0004 | < 10.67 | 76 | 56.57% to 88.50% | 76 | 56.57% to 88.50% |
| **IL-12 (p40)** | 0.8608 | 0.7554 to 0.9662 | <0.0001 | < 15.43 | 80 | 60.87% to 91.14% | 80 | 60.87% to 91.14% |
| **HGF** | 0.7616 | 0.6282 to 0.8950 | 0.0015 | < 4.362 | 68 | 48.41% to 82.79% | 72 | 52.42% to 85.72% |
| **IL-1α** | 0.688 | 0.5416 to 0.8344 | 0.0226 | < 883.7 | 64 | 44.52% to 79.75% | 60 | 40.74% to 76.60% |
| **IL-1β** | 0.688 | 0.5350 to 0.8410 | 0.0226 | < 123.4 | 72 | 52.42% to 85.72% | 72 | 52.42% to 85.72% |
| **IL-1RA** | 0.72 | 0.5792 to 0.8608 | 0.0076 | < 6200 | 64 | 44.52% to 79.75% | 60 | 40.74% to 76.60% |
| **IL-8** | 0.6672 | 0.5118 to 0.8226 | 0.0426 | > 1146 | 64 | 44.52% to 79.75% | 64 | 44.52% to 79.75% |
| **IL-10** | 0.7288 | 0.5874 to 0.8702 | 0.0055 | > 22.69 | 68 | 48.41% to 82.79% | 68 | 48.41% to 82.79% |
| **IL-2,** | 0.536 | 0.3731 to 0.6989 | 0.6624 | < 28.57 | 48 | 30.03% to 66.50% | 52 | 33.50% to 69.97% |
| **IL-2RA** | 0.5048 | 0.3425 to 0.6671 | 0.9536 | > 38.50 | 48 | 30.03% to 66.50% | 48 | 30.03% to 66.50% |
| **IL-4** | 0.6536 | 0.5003 to 0.8069 | 0.0625 | < 16.50 | 64 | 44.52% to 79.75% | 64 | 44.52% to 79.75% |
| **IL-7** | 0.5624 | 0.4004 to 0.7244 | 0.4492 | > 17.54 | 56 | 37.07% to 73.33% | 56 | 37.07% to 73.33% |
| **IL-9** | 0.6112 | 0.4530 to 0.7694 | 0.1775 | < 49.60 | 60 | 40.74% to 76.60% | 56 | 37.07% to 73.33% |
| **IL-12 (p70)** | 0.588 | 0.4283 to 0.7477 | 0.2859 | < 22.90 | 56 | 37.07% to 73.33% | 60 | 40.74% to 76.60% |
| **IL-13** | 0.6064 | 0.4454 to 0.7674 | 0.197 | < 1.063 | 56 | 37.07% to 73.33% | 56 | 37.07% to 73.33% |
| **IL-16** | 0.5536 | 0.3925 to 0.7147 | 0.5157 | < 976.7 | 52 | 33.50% to 69.97% | 52 | 33.50% to 69.97% |
| **IL-18** | 0.5024 | 0.3353 to 0.6695 | 0.9768 | < 309.3 | 44 | 26.67% to 62.93% | 44 | 26.67% to 62.93% |
| **IFN-α2** | 0.5728 | 0.4064 to 0.7392 | 0.3773 | < 4.899 | 64 | 44.52% to 79.75% | 60 | 40.74% to 76.60% |
| **MIF** | 0.568 | 0.4028 to 0.7332 | 0.4096 | > 628.5 | 52 | 33.50% to 69.97% | 52 | 33.50% to 69.97% |
| **TNF-β** | 0.6392 | 0.4823 to 0.7961 | 0.0914 | < 6.143 | 60 | 40.74% to 76.60% | 60 | 40.74% to 76.60% |

Table S1. Receiver operating characteristic (ROS) analysis of salivary cytokines in patients with Hashimoto’s disease and healthy controls. IL:1α, 1β, 1RA, 2, 2RA, 3,4, 5,6,7,8,9, 10, 12 (p40), 12 (p70), 13, 15, 16, 17, 18 – interleukin:1α, 1β, 1RA, 2, 2RA, 3,4, 5,6,7,8,9, 10, 12 (p40), 12 (p70), 13, 15, 16, 17, 18; IFN-γ - interferon- γ; IFN-α2 – interferon-α2; TNF-α, β – tumor necrosis factor α, β; HGF - hepatocyte growth factor; MIF - macrophage migration inhibitory factor*,* ND – no detectable.

|  | Plasma | | | | | | | |
| --- | --- | --- | --- | --- | --- | --- | --- | --- |
|  | AUC | 95% confidence interval | P value | Cut Off | Sensitivity% | 95% CI | Specificity% | 95% CI |
| **IL-3** | 0.8208 | 0.6922 to 0.9494 | 0.0001 | < 30.01 | 76 | 56.57% to 88.50% | 76 | 56.57% to 88.50% |
| **IFN-γ** | 0.8048 | 0.6836 to 0.9260 | 0.0002 | < 196.3 | 68 | 48.41% to 82.79% | 68 | 48.41% to 82.79% |
| **IL-5** | 0.7408 | 0.5999 to 0.8817 | 0.0035 | < 128.0 | 68 | 48.41% to 82.79% | 68 | 48.41% to 82.79% |
| **IL-6** | 0.7952 | 0.6564 to 0.9340 | 0.0003 | < 3.408 | 72 | 52.42% to 85.72% | 72 | 52.42% to 85.72% |
| **TNF-α** | 0.7952 | 0.6564 to 0.9340 | 0.0003 | < 3.408 | 72 | 52.42% to 85.72% | 72 | 52.42% to 85.72% |
| **IL-12 (p40)** | 0.6896 | 0.5376 to 0.8416 | 0.0215 | < 15.07 | 68 | 48.41% to 82.79% | 68 | 48.41% to 82.79% |
| **HGF** | 0.9744 | 0.9386 to 1.000 | <0.0001 | < 68.30 | 92 | 75.03% to 98.58% | 92 | 75.03% to 98.58% |
| **IL-1α** | 0.648 | 0.4926 to 0.8034 | 0.0727 | < 335.9 | 60 | 40.74% to 76.60% | 60 | 40.74% to 76.60% |
| **IL-1β** | 0.696 | 0.5487 to 0.8433 | 0.0175 | < 21.83 | 60 | 40.74% to 76.60% | 60 | 40.74% to 76.60% |
| **IL-1RA** | 0.6912 | 0.5437 to 0.8387 | 0.0204 | < 14.61 | 64 | 44.52% to 79.75% | 64 | 44.52% to 79.75% |
| **IL-8** | 0.896 | 0.8091 to 0.9829 | <0.0001 | > 18.13 | 84 | 65.35% to 93.60% | 84 | 65.35% to 93.60% |
| **IL-10** | 0.7824 | 0.6518 to 0.9130 | 0.0006 | > 30.01 | 68 | 48.41% to 82.79% | 68 | 48.41% to 82.79% |
| **IL-2,** | 0.6288 | 0.4713 to 0.7863 | 0.1183 | < 136.6 | 60 | 40.74% to 76.60% | 60 | 40.74% to 76.60% |
| **IL-2RA** | 0.6704 | 0.5129 to 0.8279 | 0.0388 | < 29.27 | 64 | 44.52% to 79.75% | 64 | 44.52% to 79.75% |
| **IL-4** | 0.5856 | 0.4196 to 0.7516 | 0.2992 | < 25.90 | 60 | 40.74% to 76.60% | 60 | 40.74% to 76.60% |
| **IL-7** | 0.6352 | 0.4748 to 0.7956 | 0.1011 | < 5.555 | 60 | 40.74% to 76.60% | 60 | 40.74% to 76.60% |
| **IL-9** | 0.5904 | 0.4277 to 0.7531 | 0.273 | < 56.50 | 60 | 40.74% to 76.60% | 60 | 40.74% to 76.60% |
| **IL-12 (p70)** | 0.7104 | 0.5658 to 0.8550 | 0.0107 | < 18.89 | 60 | 40.74% to 76.60% | 60 | 40.74% to 76.60% |
| **IL-13** | 0.6968 | 0.5442 to 0.8494 | 0.017 | < 10.89 | 64 | 44.52% to 79.75% | 68 | 48.41% to 82.79% |
| **IL-16** | 0.5 | 0.3374 to 0.6626 | >0.9999 | > 135.3 | 48 | 30.03% to 66.50% | 48 | 30.03% to 66.50% |
| **IL-18** | 0.5376 | 0.3694 to 0.7058 | 0,6484 | < 21.82 | 48 | 30.03% to 66.50% | 48 | 30.03% to 66.50% |
| **IFN-α2** | 0.5136 | 0.3506 to 0.6766 | 0.869 | > 21.98 | 56 | 37.07% to 73.33% | 56 | 37.07% to 73.33% |
| **MIF** | 0.512 | 0.3475 to 0.6765 | 0.8843 | < 190.8 | 40 | 23.40% to 59.26% | 40 | 23.40% to 59.26% |
| **TNF-β** | 0.7424 | 0.5902 to 0.8946 | 0.0033 | < 7.808 | 68 | 48.41% to 82.79% | 68 | 48.41% to 82.79% |

Table S2. Receiver operating characteristic (ROS) analysis of plasma cytokines in patients with Hashimoto’s disease and healthy controls. IL:1α, 1β, 1RA, 2, 2RA, 3,4, 5,6,7,8,9, 10, 12 (p40), 12 (p70), 13, 15, 16, 17, 18 – interleukin:1α, 1β, 1RA, 2, 2RA, 3,4, 5,6,7,8,9, 10, 12 (p40), 12 (p70), 13, 15, 16, 17, 18; IFN-γ - interferon- γ; IFN-α2 – interferon-α2; TNF-α, β – tumor necrosis factor α, β; HGF - hepatocyte growth factor; MIF - macrophage migration inhibitory factor*,* ND – no detectable.

|  | Saliva | | | | | | | |
| --- | --- | --- | --- | --- | --- | --- | --- | --- |
|  | AUC | 95% confidence interval | P value | Cut Off | Sensitivity% | 95% CI | Specificity% | 95% CI |
| **CCL27/CTACK** | 0.6912 | 0.5439 to 0.8385 | 0.0204 | <18.61 | 60 | 40.74% to 76.60% | 76 | 56.57% to 88.50% |
| **CXCL1/Gro-α** | 0.7792 | 0.6496 to 0.9088 | 0.0007 | < 10502 | 68 | 48.41% to 82.79% | 68 | 48.41% to 82.79% |
| **CCL2/MCP-1** | 0.5264 | 0.3615 to 0.6913 | 0.7489 | > 686.7 | 56 | 37.07% to 73.33% | 56 | 37.07% to 73.33% |
| **CCL3/MIP-1α,** | 0.5224 | 0.3576 to 0.6872 | 0.7859 | > 26.53 | 52 | 33.50% to 69.97% | 48 | 30.03% to 66.50% |
| **CCL4/MIP-1β** | 0.5176 | 0.3542 to 0.6810 | 0.831 | < 61.30 | 52 | 33.50% to 69.97% | 52 | 33.50% to 69.97% |
| **β-NGF,** | 0.5568 | 0.3931 to 0.7205 | 0.4909 | < 65.50 | 48 | 30.03% to 66.50% | 48 | 30.03% to 66.50% |
| **CCL5/RANTES** | 0.6016 | 0.4423 to 0.7609 | 0.2179 | < 3.103 | 60 | 40.74% to 76.60% | 60 | 40.74% to 76.60% |
| **SCGF-β,** | 0.54 | 0.3698 to 0.7102 | 0.6276 | > 99.50 | 52 | 33.50% to 69.97% | 52 | 33.50% to 69.97% |
| **CCL7/MCP-3** | 0.608 | 0.4494 to 0.7666 | 0.1903 | < 18.50 | 60 | 40.74% to 76.60% | 60 | 40.74% to 76.60% |
| **CCL11/Eotaxin** | 0.5168 | 0.3496 to 0.6840 | 0.8386 | < 19.73 | 44 | 26.67% to 62.93% | 44 | 26.67% to 62.93% |
| **CXCL9/MIG** | 0.5824 | 0.4214 to 0.7434 | 0.3177 | < 439.5 | 56 | 37.07% to 73.33% | 56 | 37.07% to 73.33% |
| **CXCL10/IP-10** | 0.608 | 0.4477 to 0.7683 | 0.1903 | < 7022 | 56 | 37.07% to 73.33% | 56 | 37.07% to 73.33% |
| **LIF** | 0.5992 | 0.4410 to 0.7574 | 0.229 | < 21.13 | 60 | 40.74% to 76.60% | 60 | 40.74% to 76.60% |
| **CXCL12/SDF-1α** | 0.5352 | 0.3723 to 0.6981 | 0.6695 | < 54.24 | 48 | 30.03% to 66.50% | 48 | 30.03% to 66.50% |

Table S3. Receiver operating characteristic (ROS) analysis of salivary chemokines in patients with Hashimoto’s disease and healthy controls. CCL27/CTACK - chemokine ligand 3 /monocyte chemoattractant protein*-*1, CCL3/MIP-1-α - chemokine ligands 3/macrophage inflammatory protein1-alpha, CCL4/MIP-1 β - chemokine ligands 4 /macrophage inflammatory protein1 β, β -NGF - β-nerve growth factor, CCL5/RANTES - chemokine ligand 5/regulated on activation, Normal T-cell Expressed and Secreted, SCF - stem cell factor, SCGF- β - stem cell growth factor- β, CCL7/MCP-3 - chemokine ligand 7/monocyte-chemotactic protein 3, CCL11/Eotaxin – chemokine ligand 11/Eatoksin, CCL27/CTACK – chemokine ligand 27/cutaneous T cell-attracting chemokine, CXCL1/GRO-α - chemokine (C-X-C motif) ligand 1 / growth-regulated oncogene-alpha, CXCL9/MIG - chemokine (C-X-C motif) ligand 9 /monokine induced by gamma interferon, CXCL10/IP-10 - chemokine (C-X-C motif) ligand 10/, Interferon gamma-induced protein 10, LIF - leukaemia inhibitory factor, CXCL12/SDF-1 α - chemokine (C-X-C motif) ligand 12/stromal cell-derived factor 1, ND - no detectable.

|  | Plasma | | | | | | | |
| --- | --- | --- | --- | --- | --- | --- | --- | --- |
|  | AUC | 95% confidence interval | P value | Cut Off | Sensitivity% | 95% CI | Specificity% | 95% CI |
| **CCL27/CTACK** | 0.9048 | 0.8175 to 0.9921 | <0.0001 | < 80.49 | 84 | 65.35% to 93.60% | 84 | 65.35% to 93.60% |
| **CXCL1/Gro-α** | 0.6048 | 0.4457 to 0.7639 | 0.2038 | < 20260 | 56 | 37.07% to 73.33% | 56 | 37.07% to 73.33% |
| **CCL2/MCP-1** | 0.6928 | 0.5470 to 0.8386 | 0.0194 | < 5.443 | 56 | 37.07% to 73.33% | 56 | 37.07% to 73.33% |
| **CCL3/MIP-1α,** | 0.5472 | 0.3836 to 0.7108 | 0.5671 | < 137.2 | 52 | 33.50% to 69.97% | 52 | 33.50% to 69.97% |
| **CCL4/MIP-1β** | 0.536 | 0.3738 to 0.6982 | 0.6624 | < 124.2 | 52 | 33.50% to 69.97% | 52 | 33.50% to 69.97% |
| **β-NGF,** | 0.5936 | 0.4351 to 0.7521 | 0.2563 | < 90.44 | 52 | 33.50% to 69.97% | 52 | 33.50% to 69.97% |
| **CCL5/RANTES** | 0.64 | 0.4846 to 0.7954 | 0.0896 | < 1575 | 60 | 40.74% to 76.60% | 60 | 40.74% to 76.60% |
| **SCGF-β,** | 0.5968 | 0.4364 to 0.7572 | 0.2404 | < 578.2 | 56 | 37.07% to 73.33% | 56 | 37.07% to 73.33% |
| **CCL7/MCP-3** | 0.608 | 0.4494 to 0.7666 | 0.1903 | < 18.50 | 60 | 40.74% to 76.60% | 60 | 40.74% to 76.60% |
| **CCL11/Eotaxin** | 0.5648 | 0.4018 to 0.7278 | 0.432 | < 71.78 | 56 | 37.07% to 73.33% | 56 | 37.07% to 73.33% |
| **CXCL9/MIG** | 0.6576 | 0.5015 to 0.8137 | 0.056 | < 94.26 | 64 | 44.52% to 79.75% | 64 | 44.52% to 79.75% |
| **CXCL10/IP-10** | 0.564 | 0.4008 to 0.7272 | 0.4377 | < 551.3 | 52 | 33.50% to 69.97% | 52 | 33.50% to 69.97% |
| **LIF** | 0.7216 | 0.5728 to 0.8704 | 0.0072 | < 30.35 | 68 | 48.41% to 82.79% | 68 | 48.41% to 82.79% |

Table S4. Receiver operating characteristic (ROS) analysis of plasma chemokines in patients with Hashimoto’s disease and healthy controls. CCL27/CTACK - chemokine ligand 3 /monocyte chemoattractant protein*-*1, CCL3/MIP-1-α - chemokine ligands 3/macrophage inflammatory protein1-alpha, CCL4/MIP-1 β - chemokine ligands 4 /macrophage inflammatory protein1 β, β -NGF - β-nerve growth factor, CCL5/RANTES - chemokine ligand 5/regulated on activation, Normal T-cell Expressed and Secreted, SCF - stem cell factor, SCGF- β - stem cell growth factor- β, CCL7/MCP-3 - chemokine ligand 7/monocyte-chemotactic protein 3, CCL11/Eotaxin – chemokine ligand 11/Eatoksin, CCL27/CTACK – chemokine ligand 27/cutaneous T cell-attracting chemokine, CXCL1/GRO-α - chemokine (C-X-C motif) ligand 1 / growth-regulated oncogene-alpha, CXCL9/MIG - chemokine (C-X-C motif) ligand 9 /monokine induced by gamma interferon, CXCL10/IP-10 - chemokine (C-X-C motif) ligand 10/, Interferon gamma-induced protein 10, LIF - leukaemia inhibitory factor, CXCL12/SDF-1 α - chemokine (C-X-C motif) ligand 12/stromal cell-derived factor 1, ND - no detectable.

|  | | Saliva | | | | | | | |
| --- | --- | --- | --- | --- | --- | --- | --- | --- | --- |
|  | | AUC | 95% confidence interval | P value | Cut Off | Sensitivity% | 95% CI | Specificity% | 95% CI |
| **G-CSF** | 0.7216 | | 0.5748 to 0.8684 | 0.0072 | < 515.2 | 72 | 52.42% to 85.72% | 72 | 52.42% to 85.72% |
| **VEGF** | 0.7456 | | 0.6062 to 0.8850 | 0.0029 | < 1792 | 72 | 52.42% to 85.72% | 72 | 52.42% to 85.72% |
| **TRAIL** | 0.664 | | 0.5092 to 0.8188 | 0.0467 | < 483.6 | 68 | 48.41% to 82.79% | 68 | 48.41% to 82.79% |
| **FGF** | 0.5768 | | 0.4171 to 0.7365 | 0.3517 | < 5.473 | 56 | 37.07% to 73.33% | 56 | 37.07% to 73.33% |
| **GM-CSF** | 0.5488 | | 0.3857 to 0.7119 | 0.554 | < 24.53 | 56 | 37.07% to 73.33% | 56 | 37.07% to 73.33% |
| **M-CSF** | 0.6024 | | 0.4443 to 0.7605 | 0.2143 | < 218.7 | 56 | 37.07% to 73.33% | 56 | 37.07% to 73.33% |
| **PDGF-BB** | 0.5552 | | 0.3925 to 0.7179 | 0.5032 | < 522.5 | 56 | 37.07% to 73.33% | 52 | 33.50% to 69.97% |

Table S5. Receiver operating characteristic (ROS) analysis of salivary growth factors in patients with Hashimoto’s disease and healthy controls. FGF- fibroblast growth factor, G-CSF - granulocyte colony-stimulating factor, GM-CSF - granulocyte-macrophage colony-stimulating factor, M-CSF - macrophage colony-stimulating factor, PDGF-BB - platelet-derived growth factor – BB, VEGF - vascular endothelial growth factor.

|  | | Plasma | | | | | | | |
| --- | --- | --- | --- | --- | --- | --- | --- | --- | --- |
|  | | AUC | 95% confidence interval | P value | Cut Off | Sensitivity% | 95% CI | Specificity% | 95% CI |
| **G-CSF** | 0.8288 | | 0.7139 to 0.9437 | <0.0001 | < 683.2 | 72 | 52.42% to 85.72% | 72 | 52.42% to 85.72% |
| **VEGF** | 0.6432 | | 0.4828 to 0.8036 | 0.0825 | < 190.1 | 64 | 44.52% to 79.75% | 64 | 44.52% to 79.75% |
| **TRAIL** | 0.5264 | | 0.3615 to 0.6913 | 0.7489 | > 686.7 | 56 | 37.07% to 73.33% | 56 | 37.07% to 73.33% |
| **FGF** | 0.5224 | | 0.3576 to 0.6872 | 0.7859 | > 26.53 | 52 | 33.50% to 69.97% | 48 | 30.03% to 66.50% |
| **GM-CSF** | 0.56 | | 0.3902 to 0.7298 | 0.4669 | < 101.6 | 56 | 37.07% to 73.33% | 56 | 37.07% to 73.33% |
| **M-CSF** | 0.688 | | 0.5327 to 0.8433 | 0.0226 | < 78.11 | 68 | 48.41% to 82.79% | 68 | 48.41% to 82.79% |
| **PDGF-BB** | 0.5408 | | 0.3774 to 0.7042 | 0.6208 | > 1093 | 48 | 30.03% to 66.50% | 48 | 30.03% to 66.50% |

Table S6. Receiver operating characteristic (ROS) analysis of plasma growth factors in patients with Hashimoto’s disease and healthy controls. FGF- fibroblast growth factor, G-CSF - granulocyte colony-stimulating factor, GM-CSF - granulocyte-macrophage colony-stimulating factor, M-CSF - macrophage colony-stimulating factor, PDGF-BB - platelet-derived growth factor – BB, VEGF - vascular endothelial growth factor.

Table S7. Stomatological characteristics of the HT subgroups (DMFT – Decayed, Missing, Filled Teeth, API – Approximal Plaque Index, SBI – sulcus bleeding index, PPD – periodontal pocket depth, NS – statistically insignificant).

|  |  |  |  |  |
| --- | --- | --- | --- | --- |
|  | HT, hyposalivation  n=15,  **M (min-max)** | | HT normal salivation  n=10,  **M (min-max)** | **p** |
| DMFT | 17 (14-25) | | 18 (15-23) | NS |
| API | 22.3 (1-32) | | 20.5 (0-29) | NS |
| SBI | 0.4 (0-1) | | 0.38 (0-0.9) | NS |
| PPD (mm) | 2.0 (0.7-2.5) | | 2.0 (0.5-2.2) | NS |

Table S8. Clinical characteristics of the participants of the HT subgroups (HT– Hashimoto patients, BMI – body mass index, TSH – thyroid-stimulating hormone, TPO-Ab – thyroid peroxidase antibody, TG-Ab – thyroid peroxidase antibody, SSA/Ro-Ab – anti-Sjögren’s syndrome type A antibody, SSB/La-Ab – anti-Sjögren’s syndrome type B antibody, HOMA-IR – homeostatic model assessment index, TG – triglyceride, NO – nitric oxide, UWS – unstimulated whole saliva, ND – not detectable, NS – statistically insignificant).

|  | HT, hyposalivation  n=15,  **M (min-max)** | HT normal salivation  n=10,  **M (min-max)** | p |
| --- | --- | --- | --- |
| Age (years) | 32.1 (27.8-39.8) | 33.5 (29-41.5) | NS |
| BMI (kg/m^2^) | 22.3 (19.2-24.61) | 21.4 (19.4-23) | NS |
| TSH (µU/mL) | 1.38 (0.7-2.98) | 1.5 (0.78-2.99) | NS |
| Free T4 (ng/mL) | 1.35 (0.8-1.62) | 1.21 (0.7-1.51) | NS |
| Free T3 (pg/mL) | 2.98 (2.63-4.45) | 2.94 (2.7-4.51) | NS |
| TPO-Ab (IU/mL) | 694 (254-768) | 453 (132.4-699) | NS |
| TG-Ab (IU/mL) | 295.6 (150-437) | 313 (145.8-428) | NS |
| SSA/Ro-Ab | ND | ND |  |
| SSB/La-Ab | ND | ND |  |
| Glucose (mg/dL) | 79.5 (73-87.4) | 70.2 (73.56-88.05) | NS |
| Insulin (mU/mL) | 3.56 (3-10.9) | 3.98 (3-8.5) | NS |
| HOMA-IR | 0.85 (0.47-2.1) | 0.9 (0.6-2.2) | NS |
| Witamin 25-OH D3 (ng/mL) | 40 (30-49) | 45.6 (30-50) | NS |
| TG (mg/dL) | 50 (39-85.21) | 48.7 (36-84.9) | NS |
| CRP mg/L | 0.7 (0.4-0.92) | 0.6 (0.23-0.87) | NS |
| HT duration (years) | 7.3 (6.5-8.3) | 4.1 (3.8-6.5) | **0.01** |
| Thyroid gland’s nodules | ND | ND |  |
| NO UWS (μmol/mg protein) | 0.3 (0.06-0.45) | 0.22 (9.02-0.56) | NS |

|  | Saliva | | | | |
| --- | --- | --- | --- | --- | --- |
|  | **Study group**  **normal salivation** | | **Study group**  **hyposalivation** | | P value |
|  | Mean ± SD | Median  **(min-max)** | Mean ± SD | Median  **(min-max)** |  |
| **IL-3** | 39 ± 12.88 | 37.25  (14-61) | 52.84 ± 18.68 | 52.77  (13-80) | 0.0401 |
| **IFN-γ** | 37.7 ± 11.18 | 35.81  (19.11-58.03) | 52.36 ± 14.76 | 52.39  (32.35-81.46) | 0.0163 |
| **IL-5** | 7.12 ± 3.919 | 6,1  (1.2-14.5) | 9.833 ± 3.625 | 10.35  (1.2-15.65) | 0.0762 |
| **IL-6** | 56.58 ± 19.96 | 58.66  (17-81) | 147 ± 67.16 | 112.5  (99-297) | <0.0001 |
| **TNF-α** | 13.41 ± 6.159 | 12.75  (2.133-22.83) | 17.37 ± 9.192 | 18.35  (1.28-37.39) | 0.2915 |
| **IL-12 (p40)** | 17.5 ± 3.917 | 19.5  (9.988-21) | 30.42 ± 4.604 | 31.5  (23-36.5) | <0.0001 |
| **HGF** | 5.305 ± 1.434 | 5.71  (3.063-6.973) | 5.484 ± 1.77 | 4.922  (3.08-8.694) | 0,849 |
| **IL-1α** | 683.6 ± 248.6 | 696.4  (408.2-1235) | 1301 ± 361 | 1246  (766.4-2182) | <0.0001 |
| **IL-1β** | 136.9 ± 76.66 | 135  (35.37-315) | 164.1 ± 92.93 | 164.4  (28.93-343.5) | 0.3669 |
| **IL-1RA** | 5465 ± 1700 | 5285  (3196-8181) | 7941 ± 2027 | 8436  (4011-10575) | 0.0036 |
| **IL-8** | 1018 ± 984.9 | 639.3  (24–3547) | 1357 ± 1335 | 908  (21–5426) | 0.2847 |
| **IL-10** | 24.1 ± 8.02 | 26  (8-39) | 16.65 ± 9.244 | 17  (2.235-40.22) | 0.016 |
| **IL-2** | 26 ± 8.083 | 25  (12-40) | 31.41 ± 13.36 | 29  (9.218-58) | 0.2405 |
| **IL-2RA** | 35.9 ± 10.14 | 36  (13-50) | 45.76 ± 24 | 39.19  (13-87) | 0.3788 |
| **IL-4** | 16.6 ± 5.082 | 16  (9-29) | 21.97 ± 8.245 | 20.04  (9-36) | 0.0366 |
| **IL-7** | 23.8 ± 9.684 | 20.5  (16-42) | 13.23 ± 5.427 | 14  (4.807-21.43) | 0.0007 |
| **IL-9** | 46.4 ± 14.43 | 43  (21-65) | 57.36 ± 25.32 | 57  (12.07-99) | 0.1915 |
| **IL-12 (p70)** | 24.3 ± 6.339 | 23.5  (13-33) | 25.18 ± 8.565 | 26  (9.988-36.5) | 0.8745 |
| **IL-13** | 1.319 ± 0.73 | 1.106  (0.4198-2.41) | 1.22 ± 0.7714 | 1.051  (0.1965-2.397) | 0.7088 |
| **IL-15** | 9.49 ± 5.669 | 8  (3.4-19.4) | 14.58 ± 11.51 | 11.6  (2.85-35.3) | 0.4623 |
| **IL-16** | 771.9 ± 567.7 | 717.6  (235.8-2039) | 1353 ± 516.4 | 1365  (364.4-2163) | 0.013 |
| **IL-18** | 247.1 ± 163.4 | 238  (19–569.5) | 536.9 ± 481.1 | 456.4  (8–1829) | 0.1541 |
| **IFN-α2** | 4.844 ± 1.121 | 5.027  (2.786–6.628) | 5.192 ± 2.229 | 5.253  (1.601–10.47) | 0.849 |
| **MIF** | 481 ± 308.4 | 402  (161-1032) | 930.1 ± 591.2 | 907  (119.5-2221) | 0.0475 |
| **TNF-β** | 6.736 ± 2.244 | 6,154  (2.747-10.33) | 7.401 ± 3.053 | 7,473  (1.758-11.87) | 0.6827 |

Table S9. Differences in salivary inflammatory profile in HT subjects with hyposalivation compared to normal salivary secretion. IL:1α, 1β, 1RA, 2, 2RA, 3,4, 5,6,7,8,9, 10, 12 (p40), 12 (p70), 13, 15, 16, 17, 18 – interleukin:1α, 1β, 1RA, 2, 2RA, 3,4, 5,6, 1β, 1RA, 2, 2RA, 3,4, 5,6,7,8,9, 10, 12 (p40), 12 (p70), 13, 15, 16, 17, 18; IFN-γ - interferon- γ; IFN-α2 – interferon-α2; TNF-α, β – tumor necrosis factor α, β; HGF - hepatocyte growth factor; MIF - macrophage migration inhibitory factor, ND – no detectable.

|  | Saliva | | | | |
| --- | --- | --- | --- | --- | --- |
|  | **Study group**  **normal salivation** | | **Study group**  **hyposalivation** | | P value |
|  | Mean ± SD | Median  **(min-max)** | Mean ± SD | Median  **(min-max)** |  |
| **CCL27/CTACK** | 20.9 ± 5.945 | 19.43  (12.77 – 35.61) | 21.46 ± 6.289 | 20.71  (11.53-36.62) | 0.6518 |
| **CXCL1/Gro-α** | 12405 ± 5548 | 12085  (3393-20401) | 16560 ± 7154 | 18157  (4705-30180) | 0.1963 |
| **CCL2/MCP-1** | 539.4 ± 401.1 | 442.5  (25-1106) | 853.3 ± 714.2 | 680.4  (24.52-2896) | 0.3383 |
| **CCL3/MIP-1α,** | 62.65 ± 102.8 | 30  (17.5-354) | 32.88 ± 23.63 | 23  (7.238-74.35) | 0.4202 |
| **CCL4/MIP-1β** | 63.75 ± 29.77 | 60.5  (16-129.5) | 60.38 ± 32.77 | 64  (2.401-111) | 0.9892 |
| **β-NGF,** | 59.35 ± 23.21 | 57.75  (23-95) | 77.65 ± 42.14 | 73.61  (8.457-143.3) | 0.2915 |
| **CCL5/RANTES** | 2.822 ± 1.267 | 1.267  (1.182-5.024) | 3.919 ± 1.452 | 3.907  (1.084-6.698) | 0.073 |
| **SCGF-β,** | 89.4 ± 41.85 | 75  (27-182.5) | 116.5 ± 78.73 | 103  (16.35-215) | 0.4611 |
| **CCL7/MCP-3** | 18.8 ± 4.29 | 18  (12-26) | 20.97 ± 6.171 | 20  (10.93-30.9) | 0.2777 |
| **CCL11/Eotaxin** | 24.01 ± 7.124 | 24.53  (14.89-33.93) | 20.47 ± 10.34 | 17.26  (9.54-43.23) | 0.1438 |
| **CXCL9/MIG** | 1603 ± 2301 | 775  (229.5-7798) | 2301 ± 644.8 | 466.5  (74.97-2044) | 0.2609 |
| **CXCL10/IP-10** | 11083 ± 5035 | 10496  (3970-19860) | 7763 ± 6029 | 6410  (833.7-23044) | 0.1083 |
| **LIF** | 21.6 ± 9.568 | 20.5  (10-46) | 28.4 ± 14.75 | 25  (8.214-53.5) | 0.2008 |
| **CXCL12/SDF-1α** | 50.7 ± 16.62 | 47  (26-74) | 60.2 ± 26.16 | 60.41  (13.95-101) | 0.3669 |

Table S10. Differences in salivary chemokine profile in HT subjects with hyposalivation compared to normal salivary secretion. CCL27/CTACK - chemokine ligand 3 /monocyte chemoattractant protein*-*1, CCL3/MIP-1-α - chemokine ligands 3/macrophage inflammatory protein1-alpha, CCL4/MIP-1 β - chemokine ligands 4 /macrophage inflammatory protein1 β, β -NGF - β-nerve growth factor, CCL5/RANTES - chemokine ligand 5/regulated on activation, Normal T-cell Expressed and Secreted, SCGF- β - stem cell growth factor- β, CCL7/MCP-3 - chemokine ligand 7/monocyte-chemotactic protein 3, CCL11/Eotaxin – chemokine ligand 11/Eatoksin, CCL27/CTACK – chemokine ligand 27/cutaneous T cell-attracting chemokine, CXCL1/GRO-α - chemokine (C-X-C motif) ligand 1 / growth-regulated oncogene-alpha, CXCL9/MIG - chemokine (C-X-C motif) ligand 9 /monokine induced by gamma interferon, CXCL10/IP-10 - chemokine (C-X-C motif) ligand 10/, Interferon gamma-induced protein 10, LIF - leukaemia inhibitory factor, CXCL12/SDF-1 α - chemokine (C-X-C motif) ligand 12/stromal cell-derived factor 1.

|  | Saliva | | | | |
| --- | --- | --- | --- | --- | --- |
|  | **Study group**  **normal salivation** | | **Study group**  **hyposalivation** | | P value |
|  | Mean ± SD | Median  **(min-max)** | Mean ± SD | Median  **(min-max)** |  |
| **G-CSF** | 609.2 ± 119.9 | 598.2  (478.5-894.8) | 550.7 ± 110.8 | 579.3  (344.1-778.2) | 0.3669 |
| **VEGF** | 1923 ± 1406 | 1344  (634.8-4154) | 3103 ± 1374 | 2807  (1016-6122) | 0.0413 |
| **TRAIL** | 2044 ± 1314 | 758.9  (363.9-4653) | 805.3 ± 730 | 490.8  (24.71-2303) | 0.1775 |
| **FGF** | 5.569 ± 0.7561 | 5.54  (3.863-6.72) | 5.849 ± 1.434 | 6.72  (3.717-8.09) | 0.8382 |
| **GM-CSF** | 23.57 ± 9.576 | 24.62  (5.405-41.46) | 26.96 ± 10.78 | 25.66  (11.85-50.55) | 0.5305 |
| **M-CSF** | 185.8 ± 94.75 | 185  (27.5-382) | 392.4 ± 315.6 | 324.9  (18.8-1301) | 0.0226 |
| **PDGF-BB** | 487.5 ± 190.1 | 470  (130-795) | 565.1 ± 303,6 | 560  (111-1060) | 0.5576 |

Table S11. Differences in salivary growth factor profile in HT subjects with hyposalivation compared to normal salivary secretion. FGF- fibroblast growth factor, G-CSF - granulocyte colony-stimulating factor, GM-CSF - granulocyte-macrophage colony-stimulating factor, M-CSF - macrophage colony-stimulating factor, PDGF-BB - platelet-derived growth factor – BB, VEGF - vascular endothelial growth factor.

| **Biomarker** | | **IL-6** | **IL-1** | **INF- γ** | **TNF-α** | **IL-12** |
| --- | --- | --- | --- | --- | --- | --- |
| **β1**  **(HT duration)** | Estimate | -7.564 | -132.4 | 2.117 | 2.089 | -2.573 |
|  | Standard error | 11.83 | 74.99 | 2.717 | 1.751 | 1.647 |
|  | P value | 0,5257 | <0.0001 | <0.0001 | 0.2391 | 0.1254 |
| **β2**  **(TPO-Ab)** | Estimate | -0.2258 | -1.023 | -0.05887 | -0.002365 | -0.03188 |
|  | Standard error | 0.1344 | 74.99 | 0.03088 | 0.01990 | 0.019 |
|  | P value | 0.1001 | 0.2364 | 0.0632 | 0.9059 | 0.0956 |
| **β3**  **(TG AB)** | Estimate | -0.0002092 | -0.3892 | 0.03231 | -0.05352 | 0.016 |
|  | Standard error | 0.1406 | 0.8918 | 0.04813 | 0.02082 | 0.01959 |
|  | P value | 0.9988 | 0.6647 | 0.1435 | 0.0136 | 0.4502 |
| **β4**  **(UWS flow)** | Estimate | -180.9 | -2483 | '-53.4 | -20.3 | -50.83 |
|  | Standard error | 64.02 | 406 | 14.71 | 9.480 | 8.917 |
|  | P value | 0.0071 | <0.0001 | 0.0007 | 0.0379 | <0.0001 |
| **β5**  **(CODS)** | Estimate | 26.7 | 140.7 | 1.527 | 0.7802 | 2.084 |
|  | Standard error | 11.04 | 69.99 | 2.536 | 1.634 | 1.537 |
|  | P value | 0.0197 | 0.0505 | 0.5502 | 0.6354 | 0.1821 |

Table S12. Multifactorial regression of selected salivary biomarkers in all enrolled patients. HT – Hashimoto’s disease, TPO-Ab – thyroid peroxidase antibody, TG-Ab – thyroid peroxidase antibody, UWS – unstimulated whole saliva, CODS – Clinical Oral Dryness Score, IL-6 – interleukin 6 , IL-1 – interleukin 1, INF- γ- interferon γ, TNF­-α - tumor necrosis factor, IL-12 – interleukin 12.

|  | **LOD**  **(LLOQ – ULOQ) pg/ml** | **Mean Intra-Assay**  **%CV*** | **Mean Inter-Assay**  **%CV*** |
| --- | --- | --- | --- |
| **IL-3** | 0.13  (0.1 – 2,139) | 5.0 | 3.9 |
| **IFN-γ** | 1.05  (1.57 – 25,665) | 3.1 | 3.6 |
| **IL-5** | 0.86  (0.36 – 59,499) | 2.3 | 2.3 |
| **IL-6** | 0.34  (0.38 – 6,244) | 2.2 | 3.0 |
| **TNF-α** | 1.1  ( 3.3 – 54,566) | 3.5 | 3.0 |
| **IL-12 (p40)** | 6.39  (14.7 – 240,582) | 4.5 | 2.4 |
| **HGF** | 7.1  ( 8.8 – 143,513) | 2.6 | 2.9 |
| **IL-1α** | 6.7  ( 3.7 – 61,154) | 3.5 | 4.9 |
| **IL-1β** | 0.2  (0.3 – 4,672) | 3.6 | 3.2 |
| **IL-1RA** | 3.2  (6.2 – 34,949) | 4.7 | 5.1 |
| **IL-8** | 0.4  (0.9 – 13,992) | 3.2 | 2.8 |
| **IL-10** | 0.7  (1.1 – 17,427) | 2.3 | 3.4 |
| **IL-2** | 0.8  ( 1.3 – 21,178) | 1.7 | 2.5 |
| **IL-2RA** | 1.7  ( 1.5 – 24,270) | 3.4 | 4.8 |
| **IL-4** | 0.1  (0.2 – 3,064) | 3.2 | 1.9 |
| **IL-7** | 1.2  ( 1.9- 31,475) | 2.7 | 3.9 |
| **IL-9** | 1.1  (3.6 – 31,527) | 2.6 | 7.1 |
| **IL-12 (p70)** | 0.8  (1.4 – 23,425) | 3.3 | 2.9 |
| **IL-13** | 0.2  (0.3 – 5,157) | 3.1 | 2.7 |
| **IL-16** | 0.3  ( 1.2 – 19,639) | 2.5 | 3.0 |
| **IL-18** | 0.3  (0.7 – 10,892) | 2.9 | 2.2 |
| **IFN-α2** | 0.5  (1.0 – 15,569) | 3.3 | 4.4 |
| **MIF** | 2.5  (2.7 – 44,168) | 3.4 | 4.7 |
| **TNF-β** | 0.4  ( 0.8 – 13,186) | 3.0 | 4.7 |
| **CCL27/CTACK** | 0.82  (2.1 – 15,656) | 2.7 | 5.2 |
| **CXCL1/Gro-α** | 13.5  ( 21.1 – 21,255) | 2.6 | 7.9 |
| **CCL2/MCP-1** | 0.4  (0.5 – 8,755) | 3.2 | 3.4 |
| **CCL3/MIP-1α,** | 0.1  (0.12 – 1,218) | 4.5 | 4.2 |
| **CCL4/MIP-1β** | 1.4  (1.4 – 1,439) | 3.4 | 2.5 |
| **β-NGF,** | 0.2  ( 0.5 – 7,655) | 2.9 | 3.9 |
| **CCL5/RANTES** | 4.0  ( 16.7 – 26,467) | 3.0 | 6.7 |
| **SCGF-β,** | 141.8  (82.1 – 1,345,200) | 2.3 | 3.8 |
| **CCL7/MCP-3** | 0.2  ( 0.5 - 4,899) | 4.4 | 4.2 |
| **CCL11/Eotaxin** | 0.05  (0.1- 2,281) | 4.4 | 1.2 |
| **CXCL9/MIG** | 1.4  ( 3.2 – 32,365) | 4.4 | 4.2 |
| **CXCL10/IP-10** | 1.4  ( 3.4 – 34,953) | 2.8 | 6.0 |
| **LIF** | 2.1  ( 3.9 – 53,806) | 2.5 | 4.7 |
| **CXCL12/SDF-1α** | 2.4  ( 7.5 – 9,381) | 2.2 | 5.4 |
| **G-CSF** | 3.63  (6.4 – 104,106) | 3.1 | 4.0 |
| **VEGF** | 10.1  ( 18.0 – 149,830) | 2.8 | 8.5 |
| **TRAIL** | 0.9  (1.8 – 29,188) | 3.4 | 4.5 |
| **FGF** | 2.5  ( 3.2 – 3,341) | 3.1 | 2.4 |
| **GM-CSF** | 0.2  ( 0.5 – 7,846) | 4.3 | 2.2 |
| **M-CSF** | 0.3  ( 0.8 – 12,290) | 2.4 | 3.6 |
| **PDGF-BB** | 3.0  ( 7.1 – 37,133) | 3.3 | 9.7 |

Table S13. LLOQ, ULOQ, LOD, and intra- and inter-assay precision %CV. Intra-assay %CV is derived from one representative assay. LLOQ and ULOQ are defined as the boundary standard curve points within which the performance specifications of individual standard points were met for a 10% intra-assay CV and recovery range of 70–130%. Data were generated using the magnetic workflow with the Bio-Plex Pro Wash Station. **(https://www.bio-rad.com/webroot/web/pdf/lsr/literature/Bulletin_7089.pdf).** IL:1α, 1β, 1RA, 2, 2RA, 3, 4, 5,6,7,8,9, 10, 12 (p40), 12 (p70), 13, 15, 16, 18 – interleukin:1α, 1β, 1RA, 2, 2RA, 3,4, 5,6, 1β, 1RA, 2, 2RA, 3,4, 5,6,7,8,9, 10, 12 (p40), 12 (p70), 13, 15, 16, 17, 18; IFN-γ - interferon- γ; IFN-α2 – interferon-α2; TNF-α, β – tumor necrosis factor α, β; HGF - hepatocyte growth factor; MIF - macrophage migration inhibitory factor, CCL27/CTACK - chemokine ligand 3 /monocyte chemoattractant protein-1, CCL3/MIP-1-α - chemokine ligands 3/macrophage inflammatory protein1-alpha, CCL4/MIP-1 β - chemokine ligands 4 /macrophage inflammatory protein1 β, β -NGF - β-nerve growth factor, CCL5/RANTES - chemokine ligand 5/regulated on activation, Normal T-cell Expressed and Secreted, SCF - stem cell factor, SCGF- β - stem cell growth factor- β, CCL7/MCP-3 - chemokine ligand 7/monocyte-chemotactic protein 3, CCL11/Eotaxin – chemokine ligand 11/Eatoksin, CCL27/CTACK – chemokine ligand 27/cutaneous T cell-attracting chemokine, CXCL1/GRO-α - chemokine (C-X-C motif) ligand 1 / growth-regulated oncogene-alpha, CXCL9/MIG - chemokine (C-X-C motif) ligand 9 /monokine induced by gamma interferon, CXCL10/IP-10 - chemokine (C-X-C motif) ligand 10/, Interferon gamma-induced protein 10, LIF - leukaemia inhibitory factor, CXCL12/SDF-1 α - chemokine (C-X-C motif) ligand 12/stromal cell-derived factor 1, FGF- fibroblast growth factor, G-CSF - granulocyte colony-stimulating factor, GM-CSF - granulocyte-macrophage colony-stimulating factor, M-CSF - macrophage colony-stimulating factor, PDGF-BB - platelet-derived growth factor – BB, VEGF - vascular endothelial growth factor.
